# Host Genetics Background Affects Intestinal Cancer Development Associated with High-Fat Diet-Induced Obesity and Type 2 Diabetes

**DOI:** 10.3390/cells13211805

**Published:** 2024-10-31

**Authors:** Aya Ghnaim, Kareem Midlej, Osayd Zohud, Sama Karram, Arne Schaefer, Yael Houri-Haddad, Iqbal M. Lone, Fuad A. Iraqi

**Affiliations:** 1 Department of Clinical Microbiology and Immunology, Faculty of Medicine and Health Sciences, Tel-Aviv University, Tel-Aviv 69978, Israel; ayaghnaim89@gmail.com (A.G.); kareemmidlej@mail.tau.ac.il (K.M.); osaydzohud@mail.tau.ac.il (O.Z.); iqbalzoo84@gmail.com (I.M.L.); 2 Department of Prosthodontics, Faculty of Dental Medicine, The Hebrew University-Hadassah, Jerusalem 9112102, Israel; sama.karram@mail.hujjii.ac.il (S.K.); houri.yael@mail.hujjii.ac.il (Y.H.-H.); 3 Department of Periodontology, Oral Medicine and Oral Surgery, Charité-University Medicine, 14197 Berlin, Germany; arne.schaefer@charite.de

**Keywords:** intestinal cancer developments, obesity, type 2 diabetes, host genetic background, collaborative cross mice

## Abstract

Background: Obesity and type 2 diabetes (T2D) promote inflammation, increasing the risk of colorectal cancer (CRC). High-fat diet (HFD)-induced obesity is key to these diseases through biological mechanisms. This study examined the impact of genetic background on the multimorbidity of intestinal cancer, T2D, and inflammation due to HFD-induced obesity. Methods: A cohort of 357 Collaborative Cross (CC) mice from 15 lines was fed either a control chow diet (CHD) or HFD for 12 weeks. Body weight was tracked biweekly, and blood glucose was assessed at weeks 6 and 12 via intraperitoneal glucose tolerance tests (IPGTT). At the study’s endpoint, intestinal polyps were counted, and cytokine profiles were analyzed to evaluate the inflammatory response. Results: HFD significantly increased blood glucose levels and body weight, with males showing higher susceptibility to T2D and obesity. Genetic variation across CC lines influenced glucose metabolism, body weight, and polyp development. Mice on HFD developed more intestinal polyps, with males showing higher counts than females. Cytokine analysis revealed diet-induced variations in pro-inflammatory markers like IL-6, IL-17A, and TNF-α, differing by genetic background and sex. Conclusions: Host genetics plays a crucial role in susceptibility to HFD-induced obesity, T2D, CRC, and inflammation. Genetic differences across CC lines contributed to variability in disease outcomes, providing insight into the genetic underpinnings of multimorbidity. This study supports gene-mapping efforts to develop personalized prevention and treatment strategies for these diseases.

## 1. Introduction

Colorectal cancer (CRC) is a heterogeneous disease known as colorectal adenocarcinoma, bowel cancer, colon cancer, or rectal cancer. CRC occurs in the colon (approximately 41% in the proximal colon, approximately 22% in the distal colon, and 28% in the rectum) and parts of the gastrointestinal system [1]. According to the International Agency for Research on Cancer (IARC), it was estimated that CRC accounted for 11% of all incident cancers in the world, which made it the third most common cancer in men and the second in women and is expected to cause many deaths [2]. Nonmodifiable risk factors of CRC are related to heredity and medical history [3]. The fact that up to 30% of CRC patients have a family history of the disease makes this reason one of the most actionable risk factors [4,5,6]. Finally, most CRC clustered in families reflects the interaction between environmental factors and genetic variation [7]. The diagnosis of CRC usually starts with polyp development. This noncancerous growth develops in the inner lining of the colon or rectum and can be detected and diagnosed by colonoscopy in higher prevalence in older age groups [8].

It is believed that one of the leading causes of CRC development is obesity. Obesity is an abnormal excessive accumulation of body fat primarily assessed by body mass index (BMI). Obesity and related metabolic disturbances are closely associated with pathologies that represent a significant burden to global health. Its prevalence has increased dramatically; two-thirds of the population in the United States is considered obese [9]. Obesity can lead to a variety of severe and complex diseases, such as cardiovascular diseases, diabetes, and an increased risk of cancer development [10]. In addition, excess body fat, particularly abdominal or visceral adiposity, can cause metabolic syndrome, characterized by disturbance of triglyceride, dyslipidemia, and dysglycemia [11,12,13]. Moreover, it increases the chance of insulin resistance, a significant factor in developing type 2 diabetes (T2D). Insulin resistance in obesity and diabetes is caused by a decreased insulin stimulant glucose transporter, metabolism in adipocytes and skeletal muscle, and suppression of hepatic glucose secretion [14].

A high-fat diet (HFD) contributes to the increased risk for obesity and type 2 diabetes through physiological mechanisms such as inflammation. Increased saturated fatty acid (SFA) concentrations correspond with the generation of inflammatory cytokines [15]. A considerable amount of evidence indicates that pro-inflammatory mediators contribute to the development of insulin resistance. In a mice model of diet-induced obesity, inhibiting TNF-α inhibits the development of obesity-associated insulin resistance, highlighting the importance of pro-inflammatory variables in the etiology of type 2 diabetes [16,17]. In turn, T2D causes the body’s organs to react with hyperinflammation, which hinders tissue regeneration and inflammation resolution, which accelerates the effect of cell proliferation. The relationship between BMI and CRC development was previously studied in animal models in both *APC*^min^ mice, a model for spontaneous colorectal cancer development, and mice treated with azoxymethane, a carcinogen used to induce colorectal cancer. It was observed that after feeding these mice a high-fat diet, their BMI increased and colon polyp formation [18,19]. Obesity and metabolic state are associated with inflammation and a higher risk of type 2 diabetes along with various chronic illnesses, according to epidemiological and molecular studies. Hyperglycemia associated with T2D can activate several pathways that increase inflammation, oxidative stress, and apoptosis [20]. The presence of inflammatory cytokines, including elevation in serum levels of IL-6 and TNF-ά, has been demonstrated in diabetes and obesity [21].

One way to understand the process behind complex traits is systems genetics. Instead of analyzing a single genetic disturbance, as in a transgenic mouse, Systems genetics allows for examining molecular interactions within a context most relevant to clinical traits, considering multiple genetic alterations as seen in natural populations [22]. Comparative mapping revealed that most murine genes are orthologous to those in the human genomes and that the murine model can replicate human situations [23]. Finally, earlier research combining genome-wide associations and family heredity (GWAS) has validated the critical involvement of genetic variables in the onset of colorectal cancer [24], and these findings support our research rationale and the design. The justification for this study was to explore the genetics of host susceptibility to the multimorbidity of diabetes type 2, obesity, and intestinal malignancies to a high-fat diet in a cohort of CC lines.

A previous report showed that recombinant inbred mouse lines (RIL) with genetically varied populations could serve as a basis for identifying risk factors in complicated human illnesses [25]. Recently, a mouse genetically diverse RIL, named the Collaborative Cross (CC), the next generation of mouse genetic reference population, allows time and cost-efficient mapping of target regions as quantitative trait loci (QTLs) that are responsible for the genetic variance of a specific complex trait [26]. The CC is a large panel of recombinant inbred (RI) mouse strains derived from a highly genetically diverse set of eight founders. Three of the CC strain’s founding members are descended from wild origins, and five of the mice are of inbred lines. A specific strategic breeding method was developed to create the genomes of all the CC founder strains.

Previous results and reports from our research, it was verified that various CC lines react differentially to HFD, where the way that T2D developed and progressed in the aftermath of dietary challenges differed significantly across male and female mice of the various CC strains, chow diet (CHD) and HFD. Furthermore, a result from new research by our research facility has demonstrated an impact of the host’s genetic background on the comorbidity between type 2 diabetes and obesity [27]. Finally, to study the multimorbidity of obesity and type 2 diabetes developments due to high-fat diet and intestinal cancer, we assessed CC mice that offer a distinctive and potent venue and tool as a first step in determining the part that genetic variables play underlying this multimorbidity.

## 2. Material and Methods

### 2.1. Ethical Statement

All animal trials in this study complied with the guidelines for the care and use of laboratory animals and were authorized by the Institutional Animal Care and Use Committee (IACUC) at Tel Aviv University, Israel, IDs 01-19-013 and 01-20-015.

### 2.2. Collaborative Cross-Mouse Research Population

To evaluate the formation of intestinal polyps, 357 genetically distinct CC mice from 15 distinct CC lines comprising both genders were included in the investigation. The Tel Aviv University animal facility provided the CC mice, which were grown there. The ethical guidelines kept them for temperature and humidity (21–23 °C). The use and distribution of mice across multiple trial cohorts are displayed in Supplementary Table S1A–C.

### 2.3. Study Design

When the mice are 8 weeks old, the 12-week trial starts. Some mice were fed a high-fat diet (HFD) throughout this time, whereas others were on a regular chow diet (CHD). Body weight was also measured every 2 weeks, and at the conclusion of the trial, specimen collection from the colon and small intestine, as well as a glucose tolerance test, was carried out.

### 2.4. Dietary Challenge

After weaning at the age of 4 weeks, the mice were on a conventional rodent chow diet (TD.2018SC, 18% Kcal from fat, 24% from protein, and 58% from carbs; Teklad Global, Harlan Inc., Madison, WI, USA) until they were 8 weeks old. The trials began at 8 weeks of mouse age. For the duration of the study, a set of mice used for the study were kept on CHD. On the contrary, the other set was switched to a high-fat Western diet (TD. 88137, 42.0% kcal from fat, 15.3% from protein, and 42.7% from carbs, primarily sucrose, Teklad Global, Harlan Inc., Madison, WI, USA) and kept on it for a period of 12 weeks.

### 2.5. Intraperitoneal Glucose Tolerance Test (IPGTT)

IPGTT was performed to identify diabetes type 2 cases in the two sets and identify abnormalities in the processing of glucose (measured by blood glucose [BG] levels). IPGTT was performed twice throughout the experiment (weeks 6 and 12 beginning from week 8, also known as week 0) on both the experimental and control animals. Throughout the 3 h of IPGTT, the body’s reaction to a glucose infusion was evaluated. The mice were given full access to water and denied food for 6 h (6:00–12:00 a.m.) on each occasion. After measuring blood glucose (fasting blood glucose), an intraperitoneal (IP) administration of a glucose solution (2.5 gm glucose per kilogram) was done. Throughout the next 3 h, blood glucose levels were assessed at various intervals (times 0, 15, 30, 60, 120, and 180 min after the glucose administration). Following the IPGTT evaluation, the mice were put back in their cages with unrestricted access to food and water so they could recuperate overnight.

### 2.6. Samples Collection

At the trial conclusion (when the mice reached 20 weeks of age), mice were sacrificed in a CO_2_ chamber. Mice were weighed, and tissues, including the spleen, liver, kidneys, intestines, and serum, were collected for further analysis.

### 2.7. Intraperitoneal Glucose Tolerance Analysis

The area under the curve (AUC) was calculated using the trapezoidal rule from 0 to 180 min after glucose injection to quantitatively assess glucose clearance activity. The AUC between any two time points is determined using the formula: (Time difference in minutes between sequential reads) × (Glucose level 1st time point + Glucose level 2nd time point)/2). The combined values of the AUCs for each of two points were used to get the overall AUC value of the 180-min experiment: total AUC0–180 = AUC0–15 + AUC15–30 + AUC30–60 + AUC60–120 + AUC120 [26].

### 2.8. Polyp Count in Small and Large Intestine

The mice were terminated after the 12-week trial period, and the small intestines and colon were extracted. The weight of the small and colon was determined, and the small intestines were split into three sections (SB1, 2, and 3); the three sections, in addition to the colon, were laid out on 3-mm Whitman filter paper. Methylene blue was used to stain the intestines after they had been fixed in 10% Neutral Buffered Formalin (NBF) for 24 h, as shown in Supplementary Figure S1. Binuclear then analyzed the specimens. Each of the four intestinal areas’ polyp counts were noted, as previously explained by Rudling et al. [28] and Dorman et al. [29]. Supplementary Figure S1 shows the polyp counts process of CC lines maintained on 42% HFD for 12 weeks on CHD. The polyp number was used as a trait for assessing the severity of intestinal cancer development.

### 2.9. Data Analysis

Data analysis was done using the R software (https://www.r-project.org/) platform and IBM SPSS software (https://www.ibm.com/spss). To compare the different CC mice lines dietary challenge (HFD vs. CHD) and the sex effect, we used ANOVA for the phenotypes Total AUC (min × mg/dL), percentage body weight (%∆BW), polyp count throughout the segments of intestine, and cytokines profile. In addition, we used the Spearman correlation matrix to evaluate the correlation between the different inflammatory cytokines. Finally, results were considered significant when *p*-value < 0.05.

### 2.10. Heritability

Heritability measures the fraction of phenotype variability attributed to genetic variation. Here, we used the ANOVA results to calculate the broad-sense heritability using the formula below:

H2=Vg/(Vg+Ve)

where *H*2 is the heritability, *Vg* is the genetic variance between the CC lines, and *Ve* is the environment variance. For further details about the calculation, see [26].

## 3. Results

This study assessed 357 CC mice originating from 15 genetically different CC lines. The mice were divided into four dietary challenge groups. The total number of females and males were 168 and 189, respectively, with an average of 10 mice per line in each group. Briefly, the 357 mice were maintained for 12 weeks under two different dietary challenges of HFD vs. CHD. The four groups (female HFD, CHD, and male HFD, CHD) undergo IPGTT at two different points at weeks 6 and 12 and are presented as areas under the curve (AUC) to assess the effect of diet on glucose clearance and diabetes development. Furthermore, the mice’s weight was evaluated every 2 weeks to define the impact of diet on body weight and obesity development. At the endpoint of the experiment, small and large intestines were extracted for polyp count.

### 3.1. Assessments of Glucose Clearance in CC Cohort as a Response to Diet at Different Time Points

**Figure 1:**
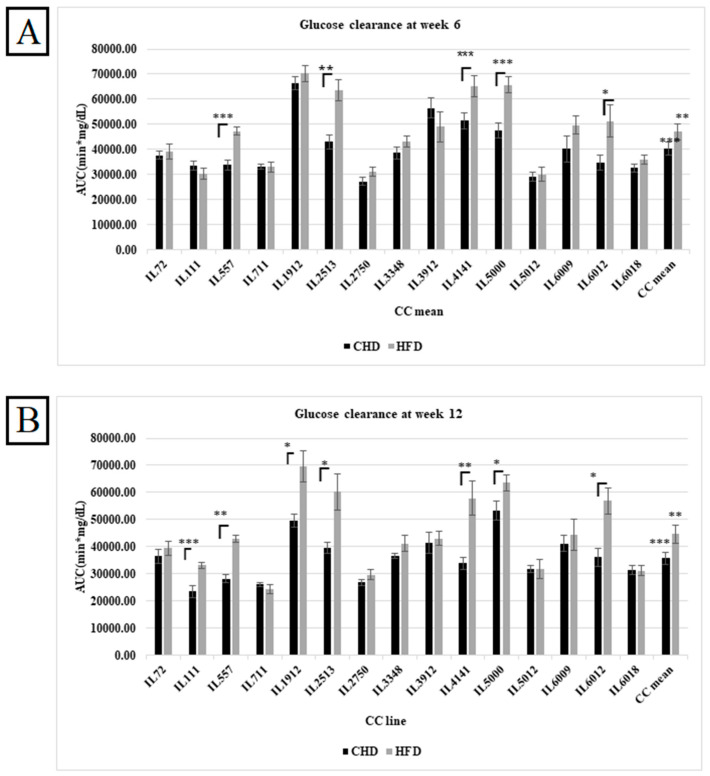
(**A**) Cumulative area under the curve (AUC; min × mg/dL) for the complete population of 15 distinct CC lines following a 6-week dietary challenge comparing HFD to CHD. The X-axis represents the various CC lines, while the Y-axis shows the AUC values. Data were assessed using an ANOVA with a significance level of *p* < 0.05. (**B**) The cumulative area under the curve (AUC; minmg/dL) for the total population of 15 different CC lines after a 6-week dietary challenge comparing HFD and CHD. The X-axis displays the different CC lines, and the Y-axis indicates the total AUC values (min × mg/dL). Data were analyzed through ANOVA with *p* < 0.05. * *p* < 0.05, ** *p* < 0.01, *** *p* < 0.005.

After weeks 6 and 12 of the experiment, an IPGTT of the different CC lines under HFD and CHD was performed for 180 min to assess the diabetogenic response. These results were converted into the total AUC of the different CC lines under HFD and CHD. The results in Figure 1A,B show the effect of HFD and CHD on glucose clearance in different CC lines at weeks 6 and 12, respectively, regardless of sex. Overall, results displayed an elevation in total AUC on HFD compared with CHD with *p*-values < 0.01, as shown in Figure 1. In week 6, five different CC lines, including IL557, IL2513, IL4141, IL5000, and IL6012, displayed a significant increase in AUC under HFD compared to CHD. These differences between the CC lines indicate the importance of the host’s genetic background in determining the response to the same stimulant.

### 3.2. Glucose Tolerance Assessments in Response to Diet and Sex at Different Time Points

**Figure 2:**
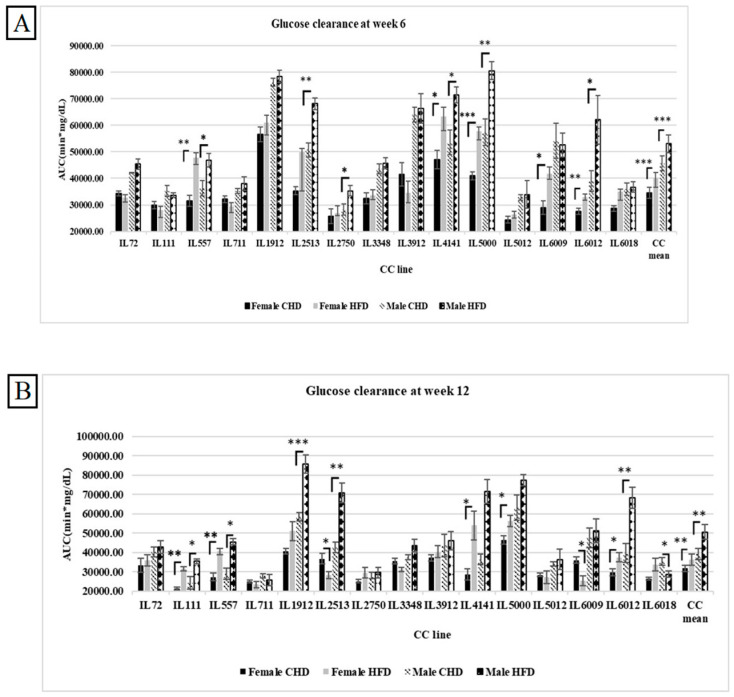
(**A**) shows the overall area under the curve (AUC; min × mg/dL) for 15 distinct CC lines after 6 weeks of dietary challenge comparing HFD to CHD in both male and female populations. The X-axis displays the various CC lines, while the Y-axis indicates the AUC values. Data were evaluated using ANOVA with *p* < 0.05. (**B**) shows the overall area under the curve (AUC; min × mg/dL) for 15 different CC lines after four months of dietary challenge comparing HFD to CHD in both male and female populations. The X-axis represents the various CC lines, and the Y-axis shows the AUC values. Data were analyzed using ANOVA with *p* < 0.05. * *p* < 0.05, ** *p* < 0.01, *** *p* < 0.005.

Our results have shown variation between the different CC lines and among both sexes as a response to HFD and CHD. As shown in Figure 2A, at week 6, the total AUC in the overall female and male populations was significantly higher in response to HFD (40,054.48 ± 3425.76 (mg/dL × min), 52,995.45 ± 3281.15 (mg/dL × min), respectively). On the other hand, the male population showed slower glucose clearance, with total AUC values higher than in the female population. This result supports the evidence that males are more susceptible to developing T2D than females. Moreover, females of five different CC lines (IL557, IL4141, IL5000, IL6009, and IL6012) showed a significant increase in total AUC values in response to HFD with *p*-values < 0.05. In contrast, CC lines (IL3912, IL72, IL111, and IL711) showed an opposite effect. Regarding the male population, five different CC lines (IL 557, IL2513, IL4141, IL5000, IL6012) showed significant elevation in total AUC values with *p*-value < 0.05 due to HFD consumption. However, in the male population, the total AUC of the same CC lines decreased in total AUC under HFD maintenance vs. CHD maintenance 52,565.62 (mg/dL × min) vs. 54,118.12 (mg/dL × min), respectively. Interestingly, after another 6 weeks under HFD, some of the CC lines of both populations behaved differently, as shown in Figure 2B. The total AUC at week 12 in the female population of lines IL2513, IL6009, and IL6018 decreased due to HFD and increased under CHD compared with the total AUC at week 6. In addition, more CC lines showed an increase in total AUC at week 12, like IL111, in both populations. These variations in glucose clearance under the same environmental factors support the impact of genetic factors besides gender impacts on T2D onset.

### 3.3. Effect of HFD on the Body Weight Changes at Two Different Time Points

**Figure 3:**
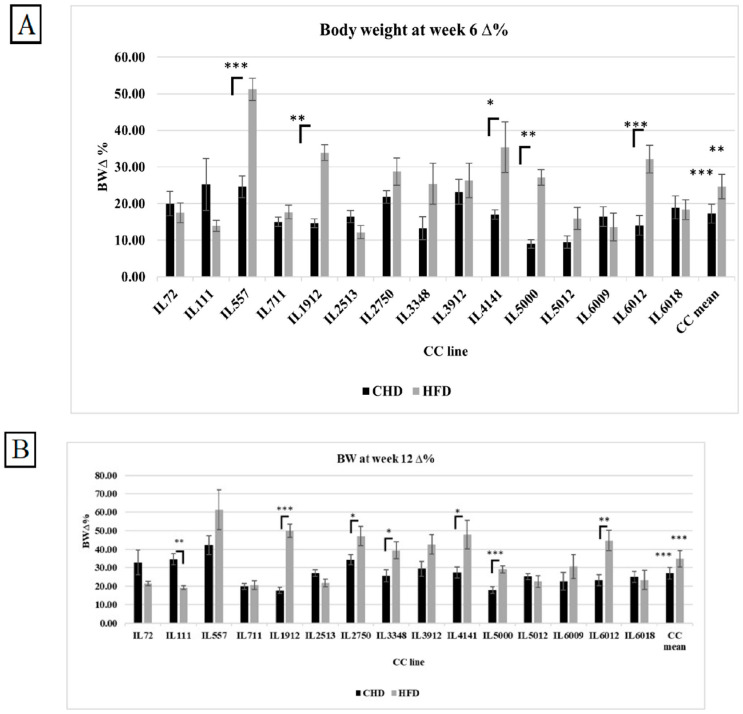
(**A**) Percentage change in body weight (%∆BW) after 6 weeks of dietary challenge (CHD vs. HFD) for a total of 15 different CC lines (X-axis). The Y-axis represents %∆BW, calculated using the formula (BW6 (g) − BW0 (g))/BW0 × 100. (**B**) Percentage change in body weight (%∆BW) after 12 weeks of dietary challenge (CHD vs. HFD) across the same 15 CC lines (X-axis). The Y-axis shows %∆BW, calculated as (BW12 (g) − BW0 (g))/BW0 × 100. * *p* < 0.05, ** *p* < 0.01, *** *p* < 0.005.

The %∆BW reflects the percentage of body weight change due to CHD and HFD consumption at two different experimental time points of 15 different CC lines, as presented in Figure 3A,B, respectively. The entire population has shown a significant increase in %∆BW on HFD compared with CHD at both experimental time points. Additionally, individual CC lines displayed different responses to HFD. On the one hand, CC lines IL557, IL1912, IL4141, IL5000, and IL6012 increased significantly in %∆BW on HFD in both weeks compared with CHD. On the other hand, IL111 revealed the opposite response. The %∆BW decreased significantly on HFD at week 12 compared with CHD (19.26 vs. 34.62, respectively). The main reason for this variation and dissimilarity between the CC lines is the genetic background of each CC line.

### 3.4. Effect of HFD and Sex on the Body Weight Changes at Two Different Time Points

**Figure 4:**
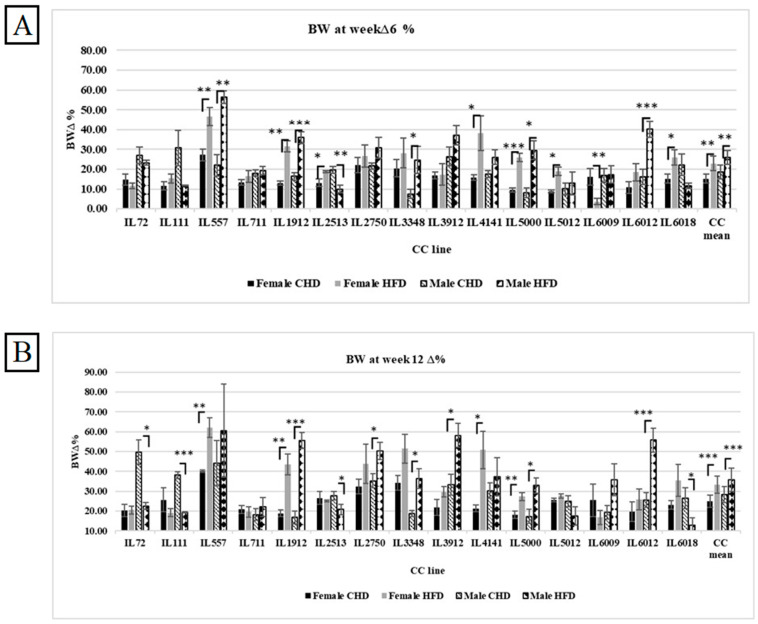
(**A**) Percent body weight change (%∆BW) after 6 weeks of dietary challenge (CHD vs. HFD) for both populations of 15 distinct CC lines (X-axis). The Y-axis shows %∆BW, calculated as (BW6 (g) − BW0 (g))/BW0 × 100. (**B**) Percent body weight change (%∆BW) after 12 weeks of dietary challenge (CHD vs. HFD) for both male and female populations of the 15 CC lines (X-axis). The Y-axis represents %∆BW, calculated as (BW12 (g) − BW0 (g))/BW0 × 100. * *p* < 0.05, ** *p* < 0.01, *** *p* < 0.005.

The results showed an effect of HFD on body weight change variability in response between the different CC lines, as well as differences among both populations. As presented in Figure 4A, both populations maintained for 6 weeks under HFD showed elevation in body weight changes compared with CHD, while the male population showed more susceptibility to HFD compared with the female population, with an average of 25.81% and 22.88%, respectively. However, some CC lines behaved differently on HFD consumption, and the body weight change decreased significantly, like the CC line IL6009, with an average of 16.15% on CHD and 3.56% on HFD. Regarding the male population, the dissimilarity between the CC lines was also observed significantly. Some lines showed a decrease in body weight change, such as line IL6018, with an average of 22.12% on CHD, 11.69% on HFD, IL111 30.67% on CHD, and vs. 11.76% on HFD. After 12 weeks, the CC mice respond strongly to HFD intake. As shown in Figure 4B, the %∆BW12 of both populations increased significantly to 33.24% in females and 35.85% in males due to HFD. Furthermore, the maintenance of the mice on HFD for an additional 6 weeks led to new results and variations between the different CC lines. For example, four CC lines in the male population, IL72, IL111, IL2513, and IL6018, declined substantially while on HFD in comparison to CHD (*p*-value < 0.05), as presented in Supplementary Table S2. In the female population, the %∆BW of IL 5012 increased significantly on HFD at week 12 than its value at week 6 (27.59% and 18.88%, respectively).

### 3.5. Intestinal Polyp Development Due to HFD Consumption in Different CC Lines

**Figure 5:**
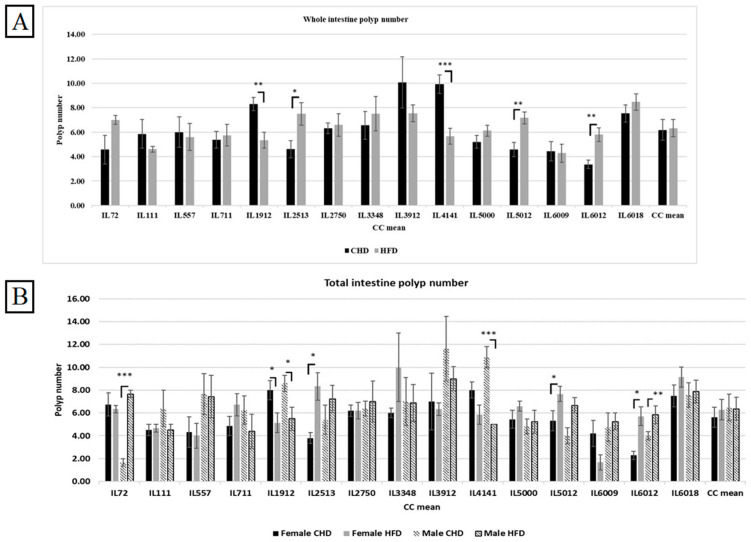
(**A**) The whole intestinal polyps count in the entire population of 15 different CC lines after being maintained for 12 weeks on HFD vs. CHD. The X-axis represents the different CC lines, and the Y-axis represents the polyp number development. Total intestinal polyps = Total small intestine polyp+ colon polyps’ number. Data was analyzed using a one-way analysis of variation (ANOVA). (**B**) Intestinal polyps count of both populations (female and male) of 15 different CC lines after being maintained for 4 months on HFD vs. CHD. The X-axis represents the different CC lines, and the Y-axis represents the polyp number development. Total intestinal polyps = Total small intestine polyp+ colon polyps’ number. Data was analyzed using ANOVA. * *p* < 0.05, ** *p* < 0.01, *** *p* < 0.005.

The sum of polyps in the small intestine and the large intestine displays the total polyps count in the whole intestine. The entire population revealed higher polys when maintained on HFD compared with CHD. As presented in Figure 5A, six different CC lines showed elevation in total intestinal polyp count, and three CC lines presented a significant increase in polyp number, IL2513, IL5012, and IL6012, with a *p*-value < 0.05. Moreover, other CC lines, IL1912 and IL4141, increased in polyp due to CHD consumption. Our results have shown variation between the different CC lines due to different diets.

### 3.6. Intestinal Polyp Development Under the Influence of Diet and Sex in Different CC Lines

Our results showed a variation in both sexes from different CC lines and in polyp development due to diet intake. Moreover, results shown in Figure 5B present an elevation in polyp count in the female population under HFD maintenance compared with CHD. However, the male population developed more polyps than females under the same diet. Additionally, we noticed the variation between the different CC lines of the same population. Within the female population, three CC lines, IL2513, IL5012, and IL6012, respond significantly to HFD and increase the polyp number with *p*-values < 0.05 compared with CHD. The results in Supplementary Table S3 present the female lines IL1912 (*p* < 0.05) and IL 6009, revealing an increase in polyp number due to CHD. The same pattern showed in the male population, where two different CC lines IL72, and IL6012, increased in polyp number due to HFD consumption, while IL1912 and IL4141 showed more polyps number on CHD than on HFD. The dissimilarity between the CC lines and among the sexes supports our idea that genetic elements influence the presence and development of CRC.

### 3.7. Small Intestinal Polyp Development Under the Influence of Diet in Different CC Lines

**Figure 6:**
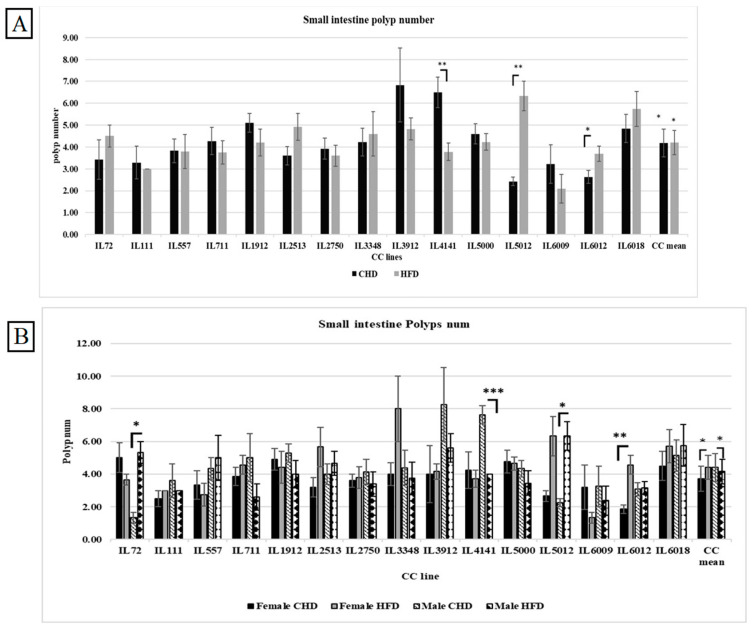
(**A**) The small intestine Polyps number among the entire population of 15 different CC lines was maintained for 12 weeks on HFD vs. CHD. The X-axis represents the different CC lines, and the Y-axis represents the polyp number development. Data was analyzed using a one-way analysis of variation (ANOVA). (**B**) Small intestine polyp number in both populations (female and male) of 15 different CC lines maintained for 12 weeks under dietary challenge (CHD vs. HFD). The X-axis represents the different CC lines, and the Y-axis represents the number of polyps in the small intestine. Data was analyzed using a one-way analysis of variation (ANOVA). * *p* < 0.05, ** *p* < 0.01, *** *p* < 0.005.

As presented in Figure 6A, the consumption of HFD in the entire population increased polyp number significantly with *p*-value < 0.01. The different CC lines behaved differently compared to HFD. In addition, some CC lines weren’t affected by diet challenges such as IL557. The fact that individual CC lines respond differently to diet under the same environmental factors confirms the importance of studying the susceptible genetic factors.

### 3.8. Small Intestinal Polyp Development Under the Influence of Diet and Sex in Different CC Lines

Figure 6B shows the polyp number in the small intestine of the different CC lines in both populations. Surprisingly, the polyp number under CHD was significantly higher in males than under HFD, with a *p*-value < 0.05. On the contrary, the female population displayed a significant polyp number in response to HFD, with a mean of 4.34 ± 0.21 (*p* < 0.05). Moreover, the variation in response to HFD across CC lines in the male cohort was revealed by three CC lines. IL72 and IL 5012 in the male cohort showed significant elevation of polyp number on HFD (5.33 ± 0.66, 6.33 ± 0.88 respectively) compared with CHD (1.33 ± 0.33, 2.25 ± 0.25 respectively). The variety of results between the different CC lines proves the genetic makeup of intestinal cancer development.

### 3.9. Colon Polyp Development Under the Influence of Diet in the Overall Population, Regardless of Sex

**Figure 7:**
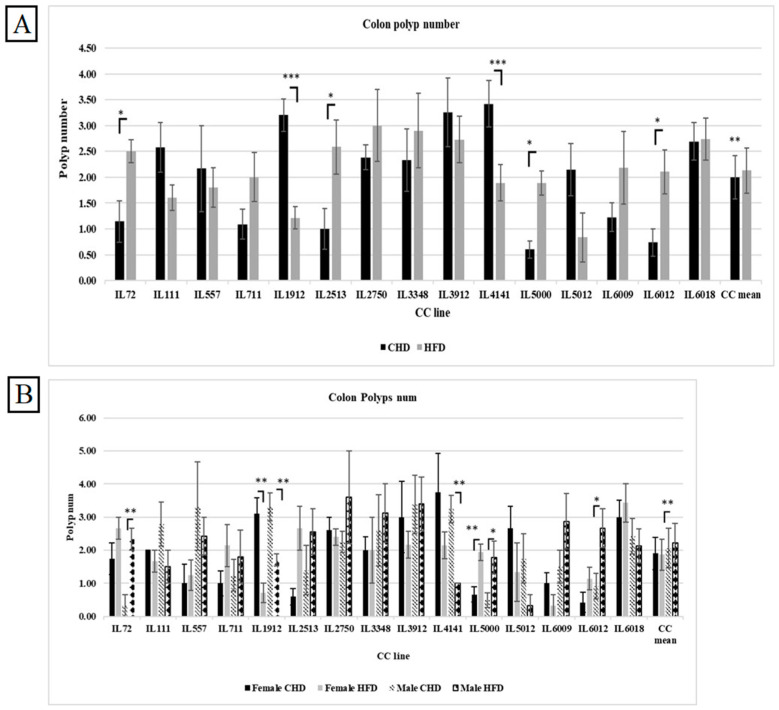
Total colon polyp number in 15 different CC lines for both population females and males, combined (**A**) and separated (**B**) maintained for four months under dietary challenge HFD (42%) vs. CHD (18%). The X-axis presents the different CC lines and the mean of the entire population; the Y-axis presents the number of polyps in the colon for both females and males. Data was analyzed using a one-way analysis of variation (ANOVA). * *p* < 0.05, ** *p* < 0.01, *** *p* < 0.005.

Our results in Figure 7A indicate an increase in polyp counts in the overall population in reaction to HFD as opposed to CHD (2.13 vs. 1.99, respectively). However, when we compared the effect of diet between the different CC lines, we found variation and dissimilarity in response to diet. As shown in Supplementary Table S4, CC lines, including IL72, IL2513, IL5000, and IL 6012, developed significantly more polyps under HFD when compared with CHD (*p* < 0.05). While CC lines IL1912 and IL4141 significantly increased the polyp number under CHD compared with HFD (*p* < 0.05). Since the environmental factors are constant, we observe a difference in response between different CC lines. These results confirm and support the role of genetic background in disease development.

### 3.10. Colon Polyp Development Under the Influence of Diet and Sex in Different CC Lines

The results showed variation in response to diet between the many CC strains among the same cohort. As shown in Figure 7B, males of the three CC lines IL72, IL5000, and IL6012 had significantly increased polyp counts in the colon on HFD maintenance with a *p*-value < 0.05. On the contrary, in the male population, the polyp number of IL4141 as well as IL1912 on CHD were highly significant than on HFD (3.25 ± 0.41, 1 ± 0.00 respectively, and 3.30 ± 0.42, 1.71 ± 0.184 respectively) with *p*-value < 0.01. Regarding the female population, IL5000 significantly developed more polyps under HFD than CHD (1.94 ± 0.25, 0.67 ± 0.23, respectively). IL1912 in the female population developed significantly more polyps on CHD than on HFD (3.10 ± 0.48, 0.71 ± 0.28, respectively) with *p*-value < 0.01. The fact that the whole experiment took place under the same environmental condition and still got the differences in response indicates that the reason for the variation is the genetic background of each CC line.

### 3.11. Assessment of Cytokines Profile of the CC Lines Under Dietary Challenge

**Figure 8:**
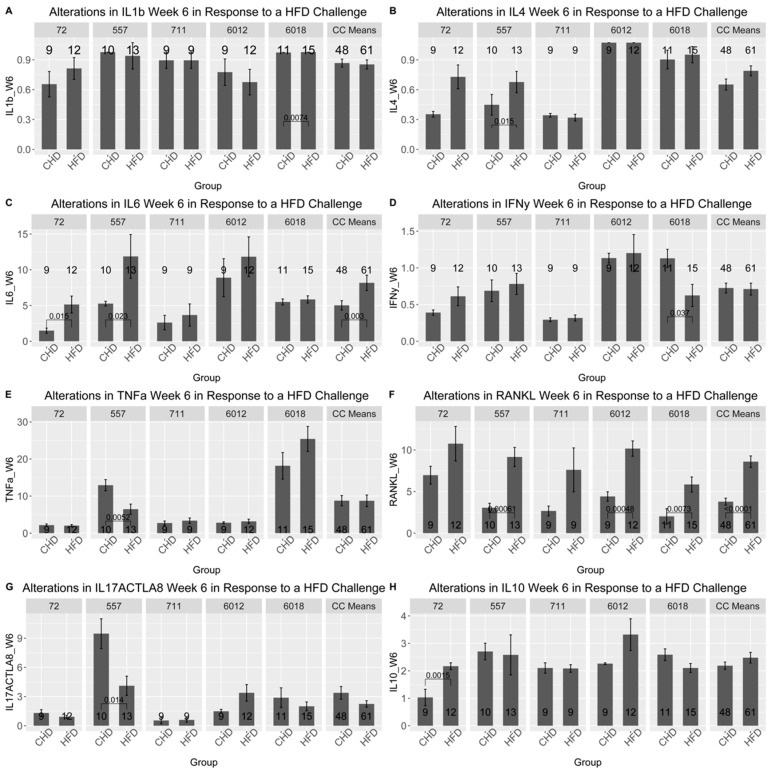
Cytokines profiles for ten different pro-inflammatory cytokines in male and female mice of 5 different CC lines at week six during the dietary challenge of either HFD (42%) or CHD (18%). The X-axis presents the different pro-inflammatory cytokines, and the Y-axis presents cytokines levels in blood serum for both female and male mice. Data was analyzed by ANOVA. (**A**–**H**) show the results of the following cytokines, IL16, IL4, IFNy, TNFa, RANKL, IL17ACTLA8, respectively.

The results of the cytokines profile were obtained after the analysis of the blood serum of five different CC lines at two different time points (at weeks 6 and week 12). As presented in Figure 8 and Figures S2–S6, the five different CC lines showed different cytokines profiles as a result of dietary challenges at weeks 6 and 12, as well as in both populations. At week 6, the RANKL cytokine in the male population of all CC lines increased significantly under HFD compared with CHD. Moreover, cytokine IL-6 significantly increased CC lines IL72, IL557 and IL711. Interestingly, CC lines IL6012 and IL6018 revealed different levels of the cytokine TNF-α as a response to HFD consumption. In contrast, the level of TNF-α increased significantly in IL6018, contrary to IL6012, which decreased substantially under HFD intake. Additionally, the CC lines showed different results over time. Under HFD, the IL-6 cytokine of CC line IL711 at week 12 decreased significantly compared with CHD. In the female population, different cytokine profiles showed different expression levels. In line IL557 maintained on HFD showed more changes in cytokine concentration over time compared with their counterparts maintained on CHD. Furthermore, this CC line displayed a substantial rise in IL1-β and IL-4 levels and a significant decline in both RANKL and IL-17A cytokine concentration due to HFD maintenance for 6 weeks. In contrast, CC line IL6012 revealed a substantial elevation of IL-17A, RANKL, and TNF-α due to HFD intake. Some CC lines showed a curve inversion after 12 weeks of the dietary challenge. In CC line 557 at week 6, a significant decrease in IL-6 and RANKL on HFD was observed. The different cytokine levels between the different CC lines confirm the importance of genetics in the development of various complex diseases that have been studied in the current research.

### 3.12. Correlation Analysis Between the Studied Phenotypes

To clarify and better understand the correlation and the co-occurrence of several medical disorders in a single instance, we aimed to investigate how the host’s genetic background affects disease multimorbidity. We focused on the development of colorectal cancer, T2D development, and obesity caused by HFD consumption. The coefficient values can range from +1 to −1, where +1 indicates a strong positive correlation, −1 indicates an entirely negative relation, and a 0 value indicates no existence of correlation. The color in the heatmap shows the level of the values of measured traits in each figure. The red color shows the highest values, the light blue color shows the lowest values, and other colors between these two extremes show intermediate values.

The heatmap in Supplementary Figure S7A represents the correlations between the tested phenotypes (Total AUC 6,12, %∆BW6,12, Total small intestine polyps, Total colon polyp count) in each experimental group (Female CHD vs. female HFD and Male CHD vs. male HFD) of the overall population for 15 different CC lines. According to the correlation matrix for the overall female population, there is a strong positive correlation between total AUC 6 and total AUC 12 under HFD with coefficient value (0.79), the same pattern seen between the body weight changes at week 6 and body weight changes at week 12 (%∆BW). Moreover, the relation between the total small intestine polyp number ranged from negative correlation to positive correlation. Interestingly, our results displayed a stronger association between the characteristics as CC lines were analyzed individually. For example, Supplementary Figure S7B showed a strong effect of HFD on multimorbidity and disease development. Under HFD, the total intestinal and colon polyps correlated significantly and strongly with total AUC 6 and %∆BW6. Moreover, this relation converted from a negative on CHD to a positive relation on HFD. The female population under HFD showed a stronger correlation than the male population under the same diet. In contrast, IL 5012 Supplementary Figure S7C. The significant variation in the correlation between the different traits and the different CC lines demonstrates the effect of genetic factors on diseases’ multimorbidities.

**Figure 9:**
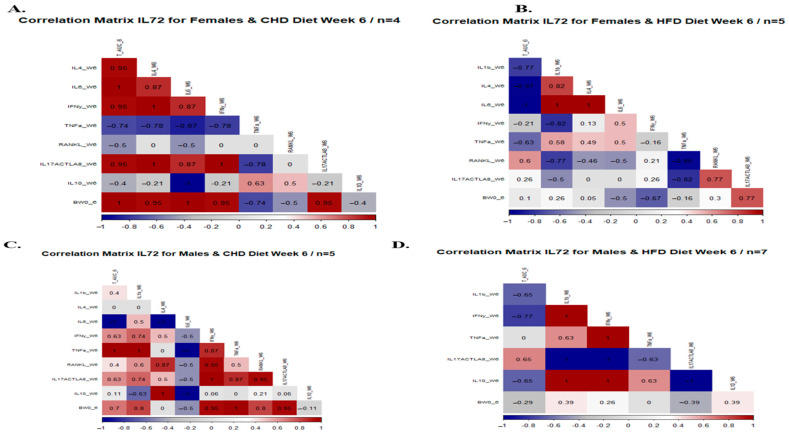
Correlation matrix between different inflammatory cytokines, % ∆BWT6, total AUC 6, for both populations (female and male). A correlation matrix of three different CC lines (IL 557, IL711, IL72) of both populations after 6 weeks of diet challenge CHD vs. HFD intake.

In the sixth week of the experiment, we analyzed the mice’s serum to determine the effect of diet on the concentration of inflammatory cytokines and its relationship with the total AUC 6 and %BW 6. For example, the maintenance of HFD strengthens the relationship between the different cytokines in the female population. As presented in Figure 9 and Supplementary Figure S8, the correlation matrix of the female population showed a conversion from a weak relation to a strong positive correlation after 6 weeks under HFD compared with CHD. Over the whole project, we focused on the response of each CC line separately due to HFD intake. Supplementary Figure S8 presented the correlation matrix between the cytokine level and phenotypes (total AUC 6 and % BW) of each CC line individually for both male and female populations under dietary challenge. The male population of CC lines IL557 and IL711 on HFD revealed a positive, strong correlation between total AUC 6, IL-6 (0.56, 0.5 respectively), and IL-10 (0.7, 0.77 respectively),

The correlation between different phenotypes and the inflammatory cytokines is presented in Supplementary Figure S9 for both populations and different CC lines. Comparing both populations, the female population showed a strong positive correlation between the %∆BW and total AUC 12. The male population revealed the effect of diet on disease development by changing the heatmap from a negative correlation under CHD to a positive correlation under HFD, as described in Supplementary Figure S9.

### 3.13. Heritability

Heritability was calculated from the results of one-way ANOVA for each of the estimated characteristics evaluated by diet only and by sex and diet. Table 1A shows the different diet traits calculated separately for diet (CHD, HFD). The heritability in total AUC displayed an estimated value larger than 0.45 under both diets. Additionally, the % ∆BWT of both weeks showed estimated values of more than 0.17 under both diets. However, the highest value shown in week 6 under HFD was 0.41. The whole intestinal polyp and colon polyp numbers revealed higher estimated values under CHD (0.311, 0.349, 0.174, respectively) compared with values under HFD (0.082, 0.042, 0.097, respectively). Furthermore, the BV showed relatively the lowest estimated values and consequently leaned toward 0 in both diets. Table 1B presents the different phenotypes calculated by sex and diet separately. The female population displayed higher values of more than 0.56 of total AUC under both diets. Similarly, in the male population, the total AUC of both tested weeks 6 and 12 during the experimental procedure revealed higher values of more than 0.511 under both diets. Additionally, both sexes maintained under HFD showed higher values of %∆BWT6–12 up to 0.33 compared with a maximum value of 0.33 under CHD. Moreover, the small intestine polyp number in the female population maintained on HFD showed higher heritability values compared with females under CHD (0.225, 0.059, respectively). In contrast, the male population’s polyps in the small intestine displayed lower heritability values under HFD than CHD (0.069, 0.249, respectively). On the other hand, our calculated results revealed higher estimated values in colon polyp numbers in both populations under CHD compared with HFD.

## 4. Discussion

Obesity is presently considered one of the leading public health concerns since it is involved in the global burden of chronic diseases, including cardiovascular diseases, diabetes type 2, and particular types of cancers [30]. Since 1980, the prevalence of obesity doubled worldwide as well, and about a third of the global population has been determined to be obese or overweight [31]. Dramatic obesity rate enhancement has been shown in both populations of males and females as well as in older individuals, especially in older women [32]. Environmental risk factors are involved in obesity development, including excessive fat intake, low physical activity, and age [33]. In addition, studies showed that around 40–70% of obesity development is caused by genetic factors, which play a crucial role in its development [34]. The GWAS identified over 400 genes associated with T2D [35,36]. However, only 5% of these genes are in obesity progression [37].

Obesity is a complex condition and one of the main reasons for T2D development. Both obesity and T2D are linked to metabolic disturbance and could contribute to chronic inflammation. These conditions are induced by maintenance on a high-fat diet (HFD). The HFD plays a vital part in the development of obesity and raises concerns about the development of T2D through various biological mechanisms. The elevation of saturated fatty acids (SFA) may produce inflammatory cytokines. Moreover, inflammation, obesity, and diabetes are associated with insulin resistance in metabolic and adipose tissue. Inflammation increases cytokine production, alters the concentration of circulated adipokines in the liver and skeletal muscle tissue, and contributes to insulin resistance [38]. Increasing insulin production from the pancreatic beta cells to maintain normal glucose levels may lead to circulating hyperinsulinemia.

Based on our previous and current results, both sexes displayed various behaviors in reaction to HFD as presented by %∆BWT shifts and level of pro-inflammatory cytokines, in addition to the formation of intestinal polyps. An increase was shown in the %∆BWT when contrasting the populations of males and females. However, different CC strains react variously to the die [33]. The variation in response among the lines further reveals the impact of the genetic makeup. The effect of diet on body weight changes stems from the genetic differences of each CC line, supported by our results, as also revealed by Abu-Toamih-Atamni et al. [26].

In this study, we assessed the blood glucose clearance using AUC measurements to identify early stages of diabetes development. Although our data indicated higher AUC values in the populations under HFD maintenance, the male population showed more significant values than the female population. These values explain that male cells take longer to absorb blood glucose and become more sensitive to diabetes than females. Furthermore, it’s essential to realize that different CC lines presented different patterns of AUC values in response to diet. The males of IL1912 on HFD showed significantly higher AUC values than IL6018, which showed substantially lower AUC values at HFD. Compared with the female population, IL4141 presented highly significant AUC values under HFD, while IL2513 showed significantly low AUC values under HFD. On the other hand, interestingly, AUC values for IL2750 males and females did not differ substantially, indicating that they were robust to the HFD exposure., as corroborated by Yosief et al. [39].

Previous studies have shown the importance of the host genetic background in developing multiple polyps. The more polyps there are, the more likely they are to possess malignant cells [40]. With an emphasis on genetic determinants, our primary objective is to use the CC model and its enormous genetic variety to discover particular genes that highlight the multimorbidity of intestinal cancer, obesity, and type 2 diabetes to be able to detect intestinal cancer at its earliest stages [41]. Our findings indicate how the 15 CC lines under study respond differently to nutritional stressors, and they have also indicated that the various host genes produce phenotypes that vary among both genders.

This study builds upon previous research by utilizing the Collaborative Cross (CC) mouse model, which offers genetic diversity unmatched by traditional inbred models. Prior studies have typically focused on specific genetic mutations or single-strain models, limiting the generalizability of their findings to broader populations. In contrast, the CC model mirrors the genetic variability observed in human populations, allowing us to explore the complex interactions between genetics, diet, and disease. This broader genetic approach enables the identification of novel loci that influence the susceptibility to obesity, type 2 diabetes (T2D), and colorectal cancer (CRC), providing a more comprehensive understanding of these diseases and their genetic underpinnings.

In addition, this research advances the study of multimorbidity by investigating how obesity, T2D, and CRC co-occur rather than examining them in isolation, as many previous studies have done. This integrated approach highlights the shared genetic and inflammatory pathways that contribute to these conditions. In particular, the study’s focus on inflammation as a key mediator between metabolic dysfunction and cancer adds a critical dimension that has been underexplored in earlier works. Additionally, by addressing sex-specific differences in disease susceptibility, this study fills an important gap in the literature, offering insights into how male and female responses to high-fat diets differ and how these differences can inform more tailored approaches to prevention and treatment.

Our findings show no discernible change in the overall polyp numbers/counts in the whole intestine due to dietary challenges. However, both the overall population and different CC lines displayed variant responses when the polyps number counted into two different intestinal segments (small intestine and colon). In the small intestine overall, the female population showed a significant increase in polyp number compared with the male population under HFD. However, in the colon segment, the polyp number remained significantly higher in the male population than in the female population under the influence of HFD consumption. This impact varies among mice with different genetic makeup. For instance, the small intestine polyp number in the male population of CC lines IL72 and IL5012 elevated under the HFD, compared with IL1414, which showed a lower polyp number under HFD than CHD. The heterogeneity in the outcome demonstrates how the host’s genetic composition affects how certain diseases develop, as corroborated earlier by Lone et al. [42].

A combination of two or more chronic disorders is known as multimorbidity, and it is prevalent among primary care patients; at least 50% of people over 50 have two or more chronic diseases [43]. Our current work provides a thorough picture of the sensitivity and resistance of these various CC lines for the diet to investigate the relationship and multimorbidity of intestinal cancer, obesity, and type 2 diabetes [43]. The heatmap analysis emphasizes the robust association between these characteristics. A noticeable association was found between the total AUC12, %∆BWT, polyp number, and part of inflammatory cytokines. At the same time, the male and female populations have different correlations in different directions. For example, a significant connection was found between total AUC12, %∆BWT, and other cytokines in the female population; the correlation was weak compared to the male population. Moreover, both populations observed a strong correlation between the colon polyp number and IL-17A cytokines. The CC mice belonging to IL711 under the effect of HFD presented more vital correlation values between total AUC, %∆BWT, and cytokines compared to other CC lines.

Our findings highlight the impact of the host genetic makeup on the progression and onset of various diseases due to HFD consumption. As we branched out and delved deeper into details in our findings, under identical environmental settings, the strains’ extreme variety and variances were noted, particularly in how each strain developed numerous chronic illnesses simultaneously. Thanks to this multimorbidity, we shall be better able to comprehend each disease’s process and its relationships with other diseases. Finding a single gene will enable us to forecast the onset of several illnesses, which allows us to stop them early.

Obesity, type 2 diabetes (T2D), and colorectal cancer (CRC) are complex disorders influenced by both genetic and environmental factors. Prior research has shown that genetic predisposition plays a significant role in these conditions, with heritability estimates for obesity-related traits ranging from 40% to 85% [44]. Genome-wide association studies (GWAS) have identified numerous candidate genes to develop these diseases, further emphasizing their polygenic nature. Our findings align with existing research demonstrating the interplay between obesity, T2D, and CRC, mainly through inflammatory pathways. Previous studies have highlighted the role of pro-inflammatory cytokines, such as IL-6 and TNF-α, as critical mediators in developing insulin resistance and obesity-induced inflammation [45]. In our study, we observed increased levels of these cytokines in response to a high-fat diet, particularly in male CC mouse lines, consistent with patterns seen in human studies on diet-induced metabolic disorders. This reinforces the well-documented role of inflammation in linking obesity and metabolic dysfunction to cancer development.Furthermore, while earlier research has often treated obesity, T2D, and CRC as separate entities, our study contributes to the growing field of multimorbidity by demonstrating how these conditions co-occur through shared genetic and inflammatory pathways. For example, we observed sex-specific differences in susceptibility to diet-induced T2D and cancer, with males showing higher vulnerability, corresponding with similar trends observed in human populations. This highlights the importance of considering sex as a critical factor in metabolic disease research, a gap that has been underexplored in previous studies.

The genetic diversity observed in the Collaborative Cross (CC) mouse model mirrors the heterogeneity found in human populations, making it highly relevant for studying the genetic basis of complex diseases such as obesity, type 2 diabetes (T2D), and colorectal cancer (CRC) [41]. Insights gained from this model can help identify potential genetic markers that may predict human disease susceptibility, paving the way for personalized medicine. These findings could inform genome-wide association studies (GWAS) in humans, focusing on specific genes and pathways linked to multimorbidity [27]. Additionally, the discovery of genetic variants influencing disease risk could contribute to the development of tailored prevention and treatment strategies based on an individual’s genetic profile. While the CC mouse model offers valuable insights, future research should aim to validate these findings in human cohorts and explore gene-environment interactions. Ultimately, this approach may also guide the design of clinical trials by considering genetic factors when stratifying patients for targeted therapies [41].

The findings from this study hold significant potential for guiding human studies, particularly in the context of clinical trials and precision medicine. The identification of key genetic variants linked to the multimorbidity of obesity, T2D, and colorectal cancer (CRC) in the CC mouse model lays the groundwork for translating these results to human populations. Future genome-wide association studies (GWAS) in humans could validate these loci, providing valuable insights for developing targeted therapies and prevention strategies. Importantly, these results highlight the need for stratifying human populations based on genetic predispositions when designing clinical interventions, especially in individuals at high risk for these conditions.

Obesity, type 2 diabetes (T2D), and colorectal cancer (CRC) are complex disorders influenced by both genetic and environmental factors [19,38]. Prior research has shown that genetic predisposition plays a significant role in these conditions, with heritability estimates for obesity-related traits ranging from 40% to 85% [44]. Genome-wide association studies (GWAS) have identified numerous candidate genes to develop these diseases, further emphasizing their polygenic nature. Our findings align with existing research demonstrating the interplay between obesity, T2D, and CRC, mainly through inflammatory pathways. Previous studies have highlighted the role of pro-inflammatory cytokines, such as IL-6 and TNF-α, as critical mediators in developing insulin resistance and obesity-induced inflammation [45]. In our study, we observed increased levels of these cytokines in response to a high-fat diet, particularly in male CC mouse lines, consistent with patterns seen in human studies on diet-induced metabolic disorders [9,21]. This reinforces the well-documented role of inflammation in linking obesity and metabolic dysfunction to cancer development.

Furthermore, while earlier research has often treated obesity, T2D, and CRC as separate entities, our study contributes to the growing field of multimorbidity by demonstrating how these conditions co-occur through shared genetic and inflammatory pathways. For example, we observed sex-specific differences in susceptibility to diet-induced T2D and cancer, with males showing higher vulnerability, corresponding with similar trends observed in human populations [20,32,33]. This highlights the importance of considering sex as a critical factor in metabolic disease research, a gap that has been underexplored in previous studies.

## 5. Conclusions

This study leverages the CC mouse model to explore the genetic underpinnings of multimorbidity involving obesity, type 2 diabetes (T2D), and colorectal cancer (CRC), providing valuable insights into the shared genetic and inflammatory pathways driving these conditions. The findings emphasize the role of genetic diversity in disease susceptibility and pave the way for identifying biomarkers that could predict disease risk in human populations. The next critical step is to validate these results in human cohorts through genome-wide association studies (GWAS) and other genetic screening approaches. This will help bridge the gap between animal models and human applications, ultimately advancing personalized therapeutic strategies.

Incorporating genetic profiling into clinical practice based on this research could enable more targeted and effective treatments for individuals predisposed to obesity-related diseases. These insights lay the groundwork for stratifying patients in clinical trials based on their genetic background, improving precision in prevention and treatment strategies. As such, this work marks an essential step towards the broader application of personalized medicine in managing metabolic and cancer-related diseases.

## Figures and Tables

**Table 1:** (**A**) Heritability values for all tested traits (%∆BW6–12, total AUC6–12), whole intestinal polyp number, small intestine polyp number, colon polyp number, and alveolar bone volume for both diets. Heritability was calculated from the ANOVA results using the formula H2=Vg/(Vg+Ve). The values range between (−1, 1). (**B**). Heritability values for all tested traits (%∆BW6–12, total AUC6–12), whole intestinal polyp number, small intestine polyp number, colon polyp number, and alveolar bone volume for both diets and sexes separately. Heritability was calculated by the ANOVA results using the formula H2=Vg/(Vg+Ve). The values range between (−1, 1).

(**A**)						
**Heritability**						
**Trait**			**CHD**		**HFD**	
**Total AUC 6**			**0.598**		**0.459**	
**Total AUC 12**			**0.465**		**0.483**	
**%∆BW6**			**0.177**		**0.419**	
**%∆BW12**			**0.236**		**0.292**	
**Whole Intestinal polyp number**.			**0.311**		**0.082**	
**Small intestine polyp number**			**0.174**		**0.097**	
**Colon polyp number**.			**0.349**		**0.042**	
(**B**)						
	**Heritability**					
	**Female**			**Male**		
**Trait**	**CHD**	**HFD**		**CHD**		**HFD**
**Total AUC 6**	**0.773**	**0.617**		**0.733**		**0.559**
**Total AUC 12**	**0.745**	**0.564**		**0.511**		**0.725**
**%∆BW6**	**0.314**	**0.412**		**0.194**		**0.572**
**%∆BW12**	**0.242**	**0.457**		**0.336**		**0.308**
**Small intestine polyp number**	**0.059**	**0.225**		**0.249**		**0.069**
**Colon polyp number**.	**0.430**	**0.238**		**0.258**		**0.013**

## Data Availability

All data shown in this work is publicly accessible.

## Supplementary Materials

The following supporting information can be downloaded at: https://www.mdpi.com/article/10.3390/cells13211805/s1, Figure S1: The process steps are extracting, washing, and preparing the small and large intestines for staining. Pictures in (1) After extracting the intestine from the mouse, (2) The Intestines after washing with Phosphate buffered saline (PBS), the Small intestine divided into 3 segments: SB1-proximal, SB2-middle, and SB3 distal and colon left uncut, and (3) The intestine after staining with methylene blue. We assume that the blue dots represent developed polyps; Figure S2: Cytokines profiles for 10 different proinflammatory cytokines in male and female mice of CC line 557 at weeks 6 and 12 during the dietary challenge of either HFD (42%) or CHD (18%). The X-axis presents the different proinflammatory cytokines, and the Y-axis presents cytokines levels in blood serum for both female and male mice. Data was analyzed by One-way Analysis of Variation (ANOVA); Figure S3 Cytokines profiles for 10 different proinflammatory cytokines in male and female mice of CC line 711 at weeks 6 and 12 during the dietary challenge of either HFD (42%) or CHD (18%). The X-axis presents the different proinflammatory cytokines, and the Y-axis presents cytokines levels in blood serum for both female and male mice. Data was analyzed by One-way Analysis of Variation (ANOVA); Figure S4: Cytokines profiles for 10 different proinflammatory cytokines in male and female mice of CC line 6012 at weeks 6 and 12 during the dietary challenge of either HFD (42%) or CHD (18%). The X-axis presents the different proinflammatory cytokines, and the Y-axis presents cytokines levels in blood serum for both female and male mice. Data was analyzed by One-way Analysis of Variation (ANOVA); Figure S5: Cytokines profiles for 10 different proinflammatory cytokines in male and female mice of CC line 6018 at weeks 6 and 12 during the dietary challenge of either HFD (42%) or CHD (18%). The X-axis presents the different proinflammatory cytokines, and the Y-axis presents cytokines levels in blood serum for both female and male mice. Data was analyzed by One-way Analysis of Variation (ANOVA); Figure S6: Cytokines profiles for 10 different proinflammatory cytokines in male and female mice of 5 different CC lines at week 12 during the dietary challenge of either HFD (42%) or CHD (18%). The X-axis presents the different proinflammatory cytokines, and the Y-axis presents cytokines levels in blood serum for both female and male mice. Data was analyzed by One-way Analysis of Variation (ANOVA); Figure S7 Correlation matrix between the different traits (Total AUC 6+ total AUC 12, %∆BW6+12, polyps’ number in small intestine and polyps’ number in colon) of the entire populations’ female and male under dietary challenge CHD Vs HFD (7A). As well as different CC lines individually (7B-17); Figure S8: Correlation matrix between different inflammatory cytokines, % ∆BWT6, total AUC 6, for both populations Female and Male. A correlation matrix of three different CC lines (Total mouse population, IL 557, IL711, IL72, and total population) of both populations after 6 weeks of diet challenge CHD Vs. HFD intake.; Figure S9: Correlation matrix between different inflammatory cytokines, % ∆BWT12, total AUC 12, whole intestine polyps, small intestine polyps, colon polyps’ number, and BV for both populations Female and Male. A correlation matrix of three different CC lines (Total mouse population, IL 557, IL711, IL72) of both populations after 12 weeks of diet challenge CHD Vs. HFD intake; Table S1: (A) Summary table shows the list of the used CC lines divided into groups (CHD, HFD) as well as the number of mice of each sex (Female and Male) in small intestine and colon polyp number studies. (B) The summary table shows the list of the used CC lines divided into groups (CHD, HFD) as well as a number of mice of each sex (Female and Male) for Micro CT analysis for PD assessment. (C) The summary table shows the list of the used CC lines that were divided into diet groups and the number of mice of each sex (Female and Male) for the correlation matrix of all the traits analysis between the six different traits; Table S2: The body weight changes (%∆BW) resulted in four different CC lines in the male population due to HFD maintenance for two other time points at weeks 6 and 12 of the experiment; Table S3: Polyps count ± SE in whole intestine parts of the female population in three different CC lines with a *p* value < 0.05; Table S4: Represent colon polyps’ number in 6 CC lines under dietary challenge CHD Vs. HFD.
