# Supplementary material for: Host Genetics Background Affects Intestinal Cancer Development Associated with High-Fat Diet-Induced Obesity and Type 2 Diabetes
Source: Cells. 2024 Oct 31;13(21):1805. doi: 10.3390/cells13211805 (PMC11545189; doi:10.3390/cells13211805)
Supplement: Supplementary file 1 [file cells-13-01805-s001.zip › cells-3218836-supplementary.pdf]

Supplementary Figure 1. The process steps are extracting, washing, and preparing the small and large intestines for staining. Pictures in (1) After extracting the intestine from the mouse, (2) The Intestines after washing with Phosphate buffered saline (PBS), the Small intestine divided into 3 segments: SB1-proximal, SB2-middle, and SB3 distal and colon left uncut, and (3) The intestine after staining with methylene blue. We assume that the blue dots represent developed polyps.

1 2 3

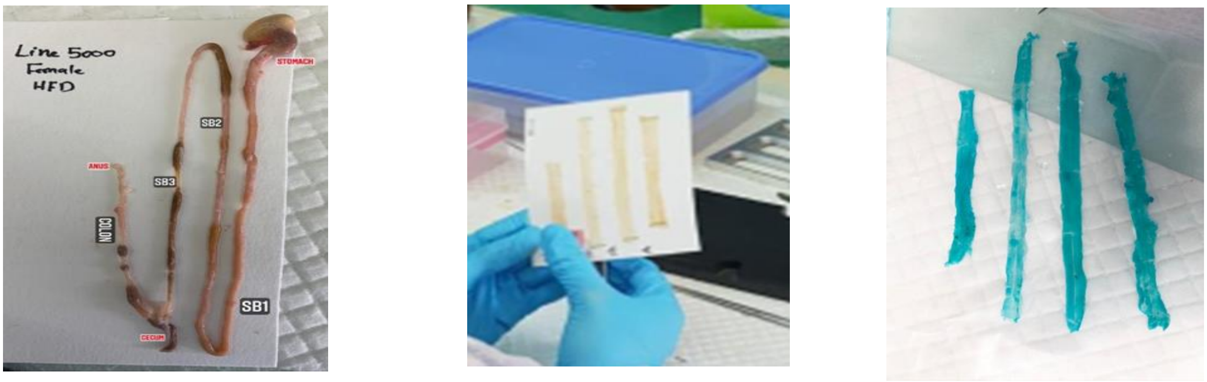

Supplementary Figure 2. Cytokines profiles for 10 different proinflammatory cytokines in male and female mice of CC line 557 at weeks 6 and 12 during the dietary challenge of either HFD (42%) or CHD (18%). The X-axis presents the different proinflammatory cytokines, and the Y-axis presents cytokines levels in blood serum for both female and male mice. Data was analyzed by One-way Analysis of Variation (ANOVA)

Cytokines profile of CC line 557 at both weeks 6 and 12

B.

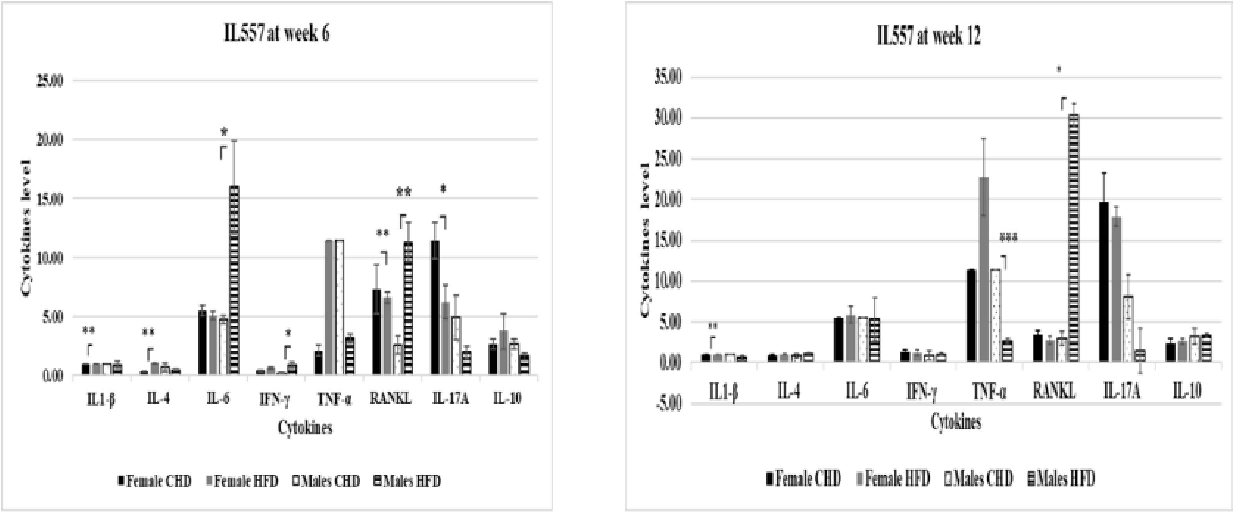

Supplementary Figure 3. Cytokines profiles for 10 different proinflammatory cytokines in male and female mice of CC line 711 at weeks 6 and 12 during the dietary challenge of either HFD (42%) or CHD (18%). The X-axis presents the different proinflammatory cytokines, and the Y-axis presents cytokines levels in blood serum for both female and male mice. Data was analyzed by One-way Analysis of Variation (ANOVA)

Cytokines profile of CC line 711 at both weeks 6 and 12

C.

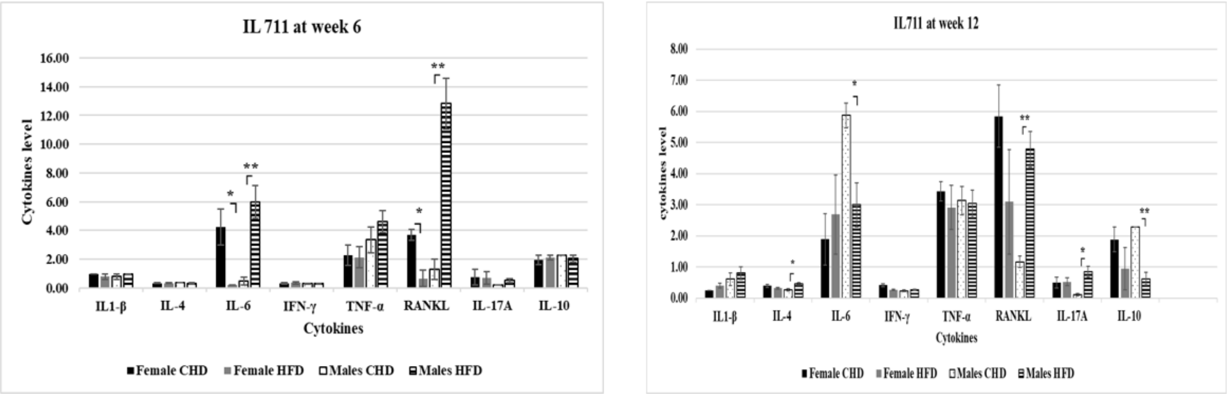

Supplementary Figure 4. Cytokines profiles for 10 different proinflammatory cytokines in male and female mice of CC line 6012 at weeks 6 and 12 during the dietary challenge of either HFD (42%) or CHD (18%). The X-axis presents the different proinflammatory cytokines, and the Y-axis presents cytokines levels in blood serum for both female and male mice. Data was analyzed by One-way Analysis of Variation (ANOVA)

D.

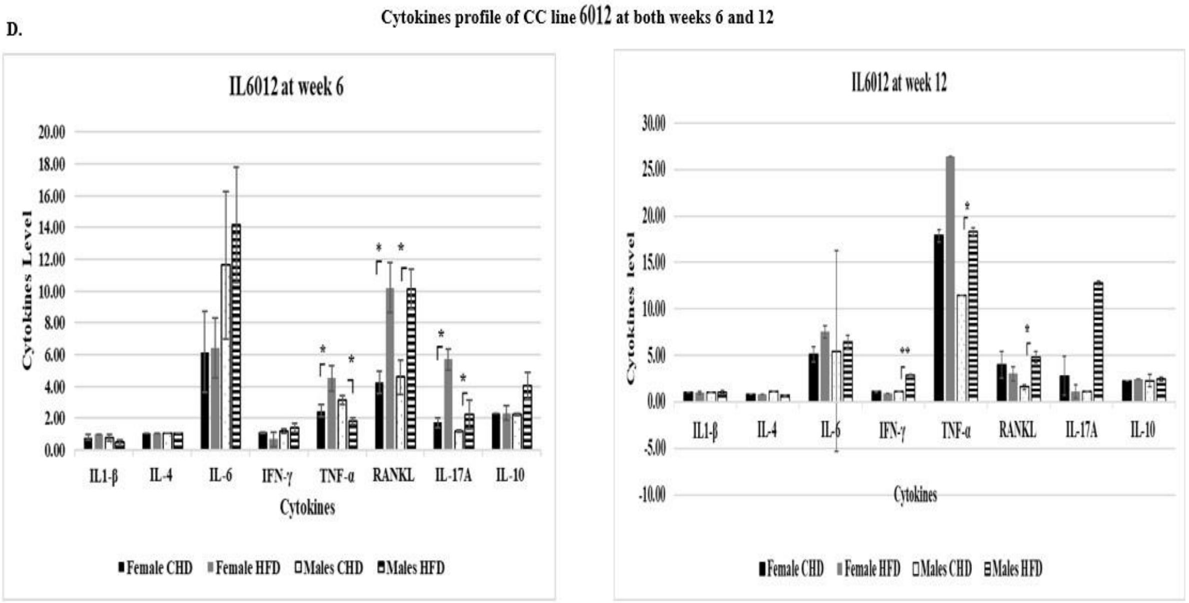

Supplementary Figure 5. Cytokines profiles for 10 different proinflammatory cytokines in male and female mice of CC line 6018 at weeks 6 and 12 during the dietary challenge of either HFD (42%) or CHD (18%). The X-axis presents the different proinflammatory cytokines, and the Y-axis presents cytokines levels in blood serum for both female and male mice. Data was analyzed by One-way Analysis of Variation (ANOVA).

Cytokines profile of CC line 6018 at both weeks 6 and 12

E.

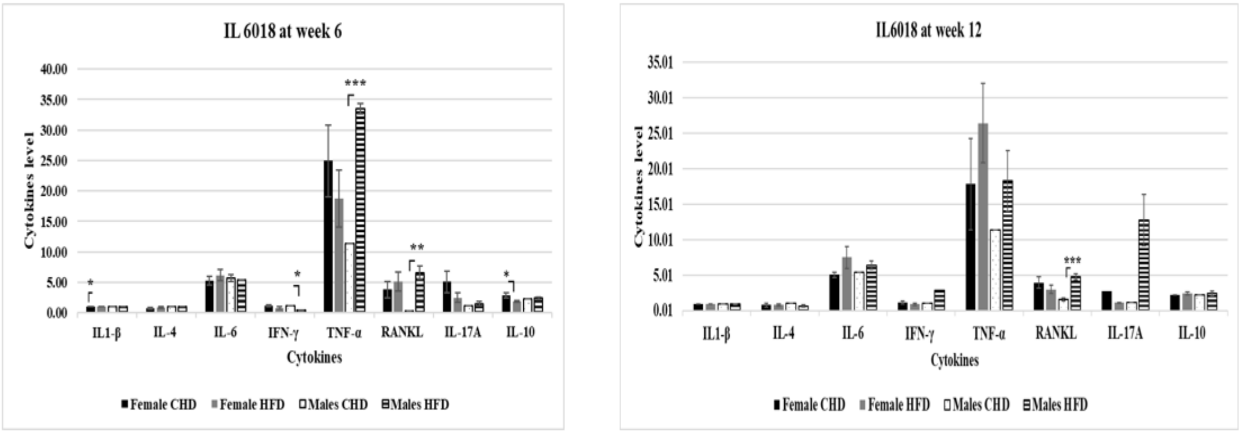

Supplementary Figure 6: Cytokines profiles for 10 different proinflammatory cytokines in male and female mice of 5 different CC lines at week 12 during the dietary challenge of either HFD (42%) or CHD (18%). The X-axis presents the different proinflammatory cytokines, and the Y-axis presents cytokines levels in blood serum for both female and male mice. Data was analyzed by One-way Analysis of Variation (ANOVA)

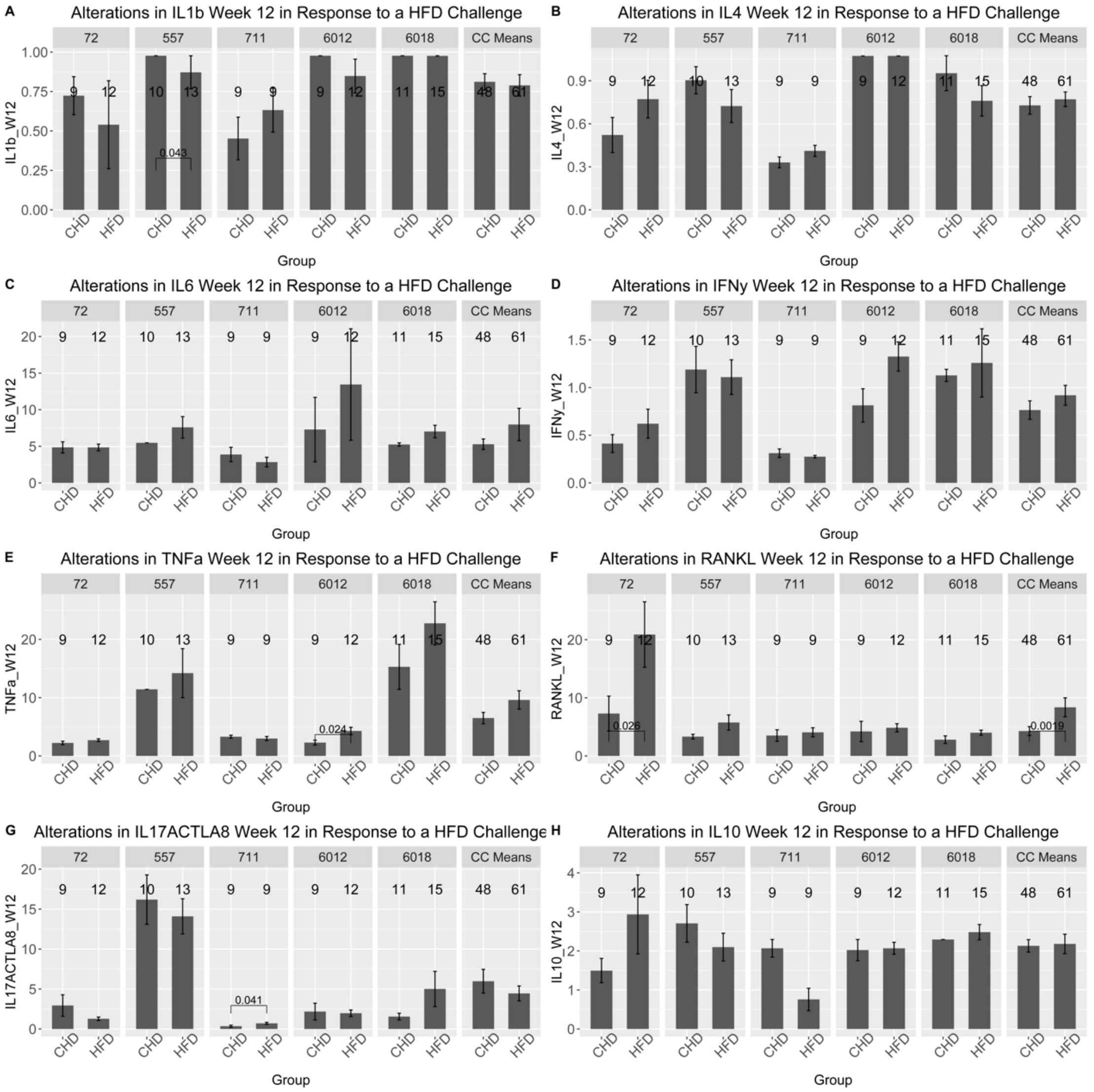

Supplementary Figure 7. Correlation matrix between the different traits (Total AUC 6+ total AUC 12, %ΔBW6+12, polyps' number in small intestine and polyps' number in colon) of the entire populations' female and male under dietary challenge CHD Vs HFD (7A). As well as different CC lines individually (7B-17).

A. The overall populations of both female and male under dietary challenge HFD Vs. CHD

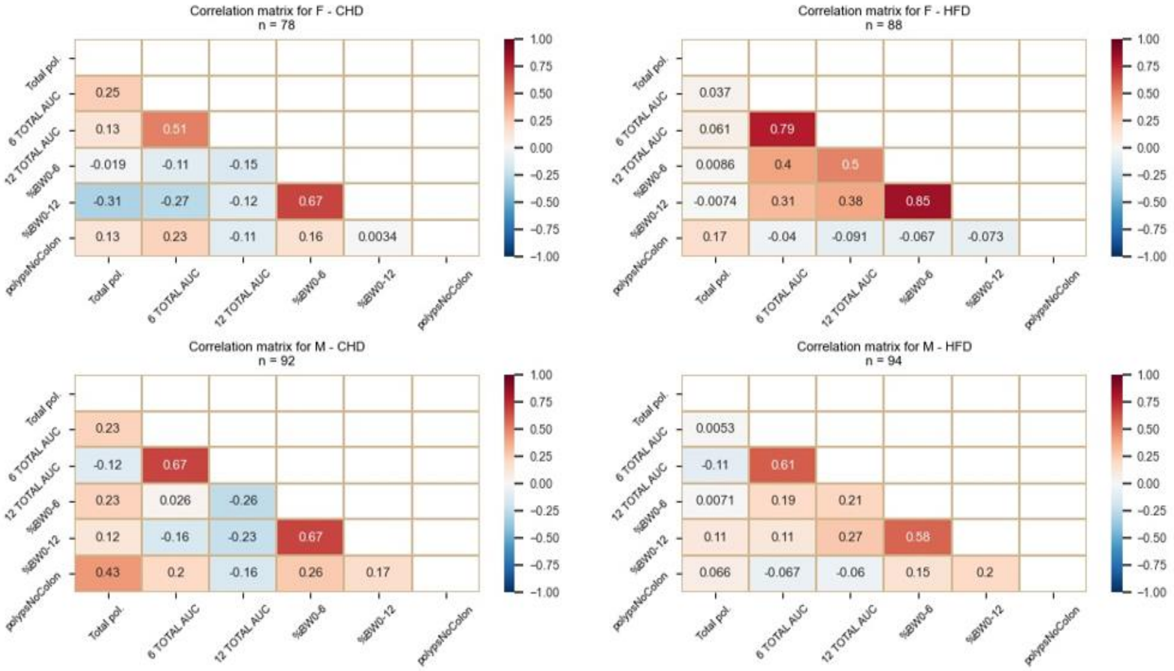

B. CC line 6009

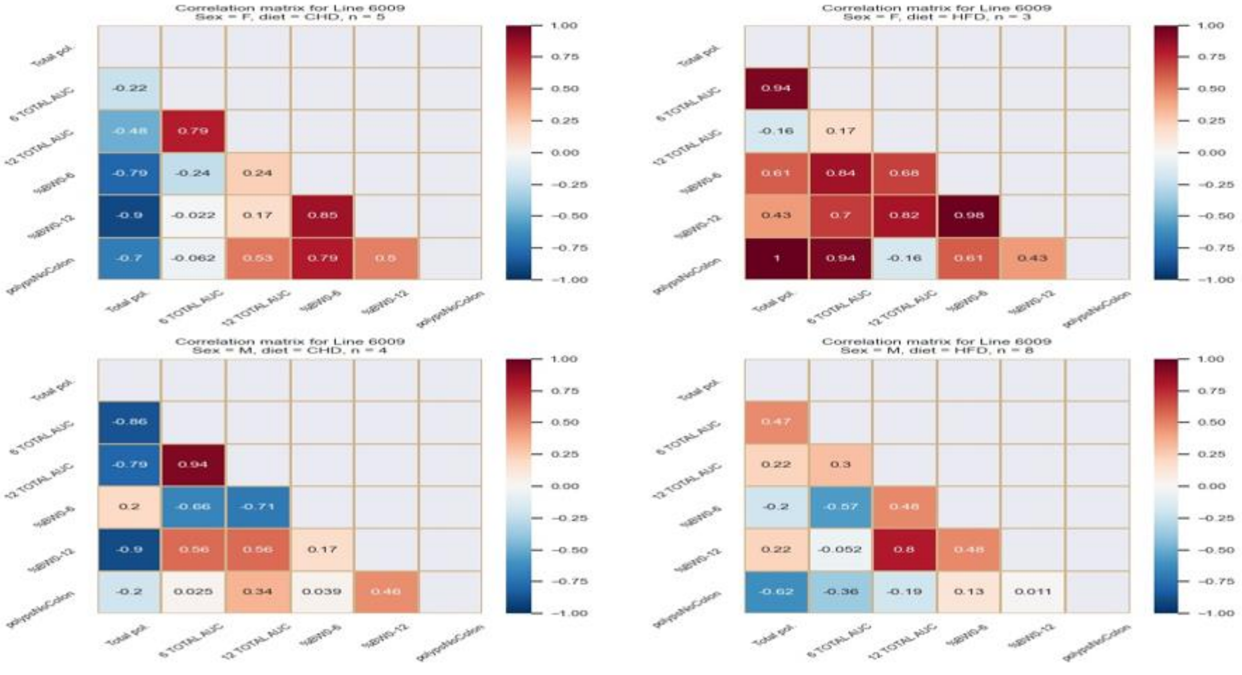

C. CC line 5012

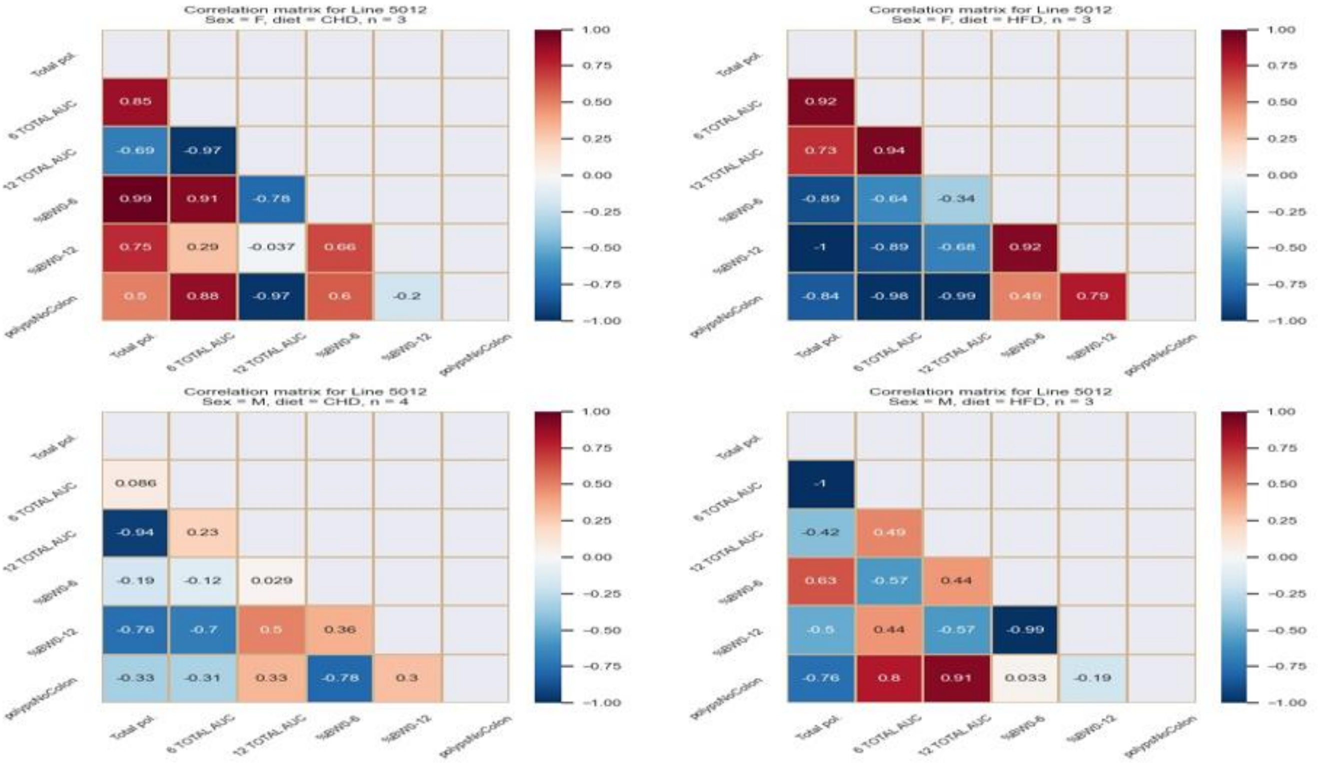

D. CC line 72

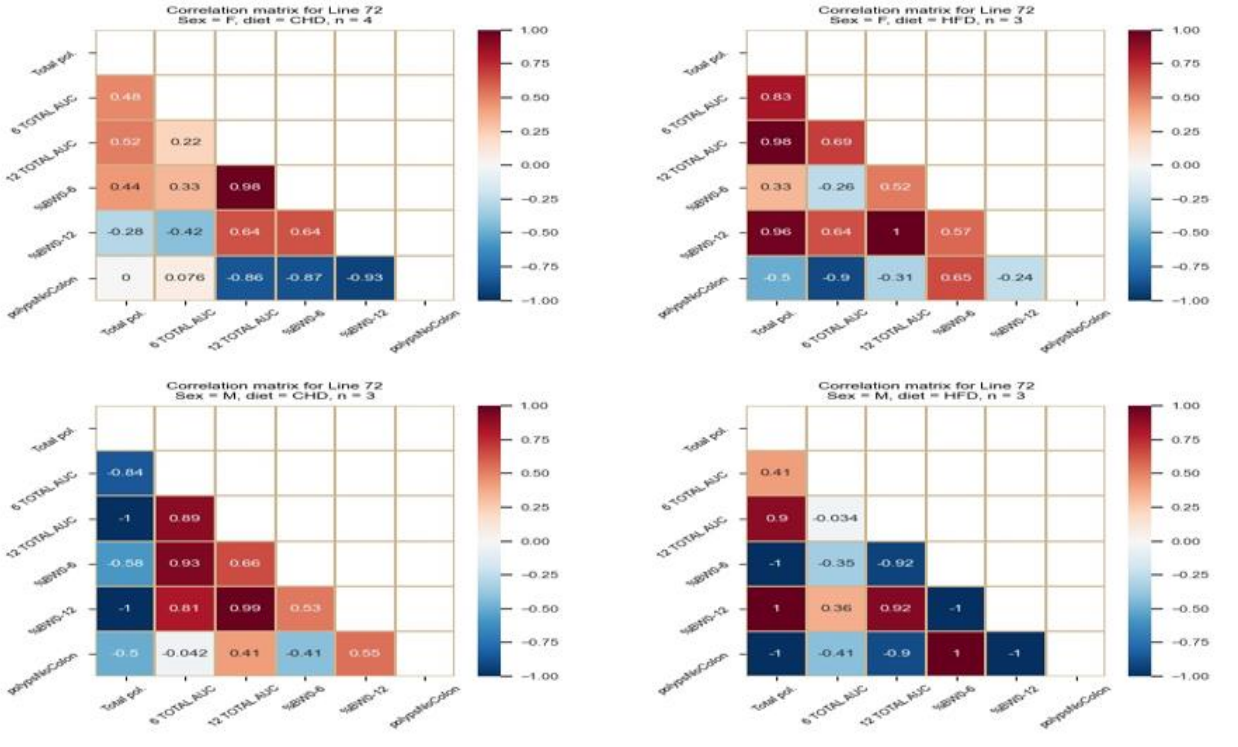

Supplementary Figure 8. Correlation matrix between different inflammatory cytokines, % ΔBWT6, total AUC 6, for both populations Female and Male. A correlation matrix of three different CC lines (Total mouse population, IL 557, IL711, IL72, and total population) of both populations after 6 weeks of diet challenge CHD Vs. HFD intake.

<u>Total mouse population,,,,,,,,,,,,where is for IL711</u>

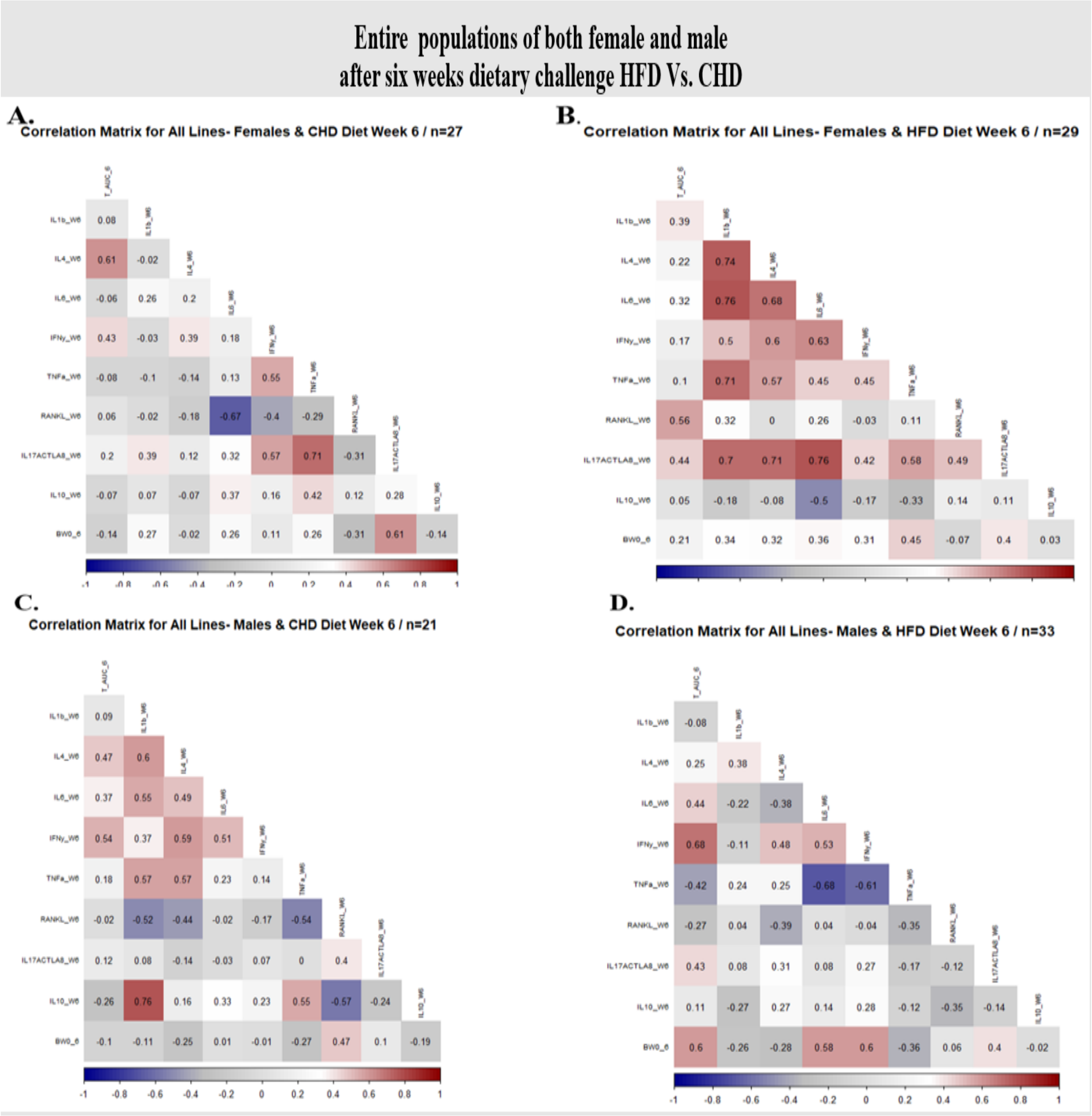

Line IL557

A. Correlation Matrix IL557 for Females & CHD Diet Week 6 / n=7

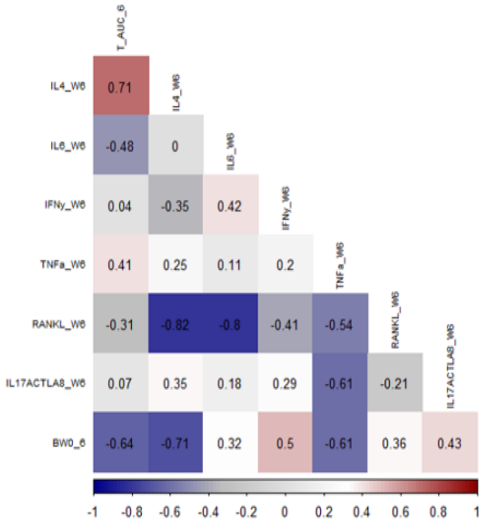

B. Correlation Matrix IL557 for Females & HFD Diet Week 6 / n=6

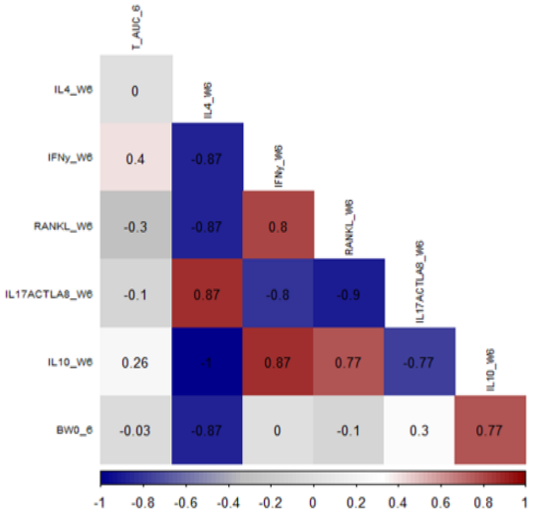

C. Correlation Matrix IL557 for Males & CHD Diet Week 6 / n=3

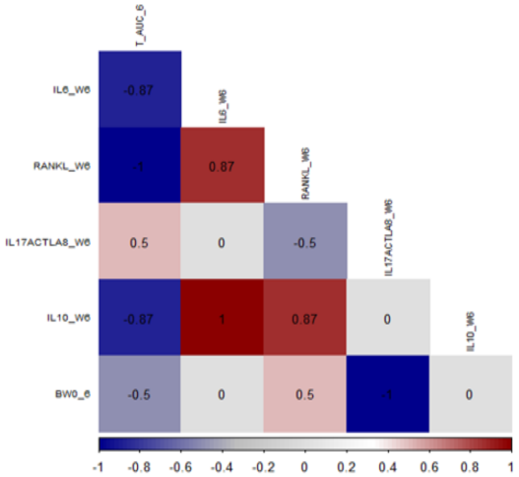

D. Correlation Matrix IL557 for Males & HFD Diet Week 6 / n=7

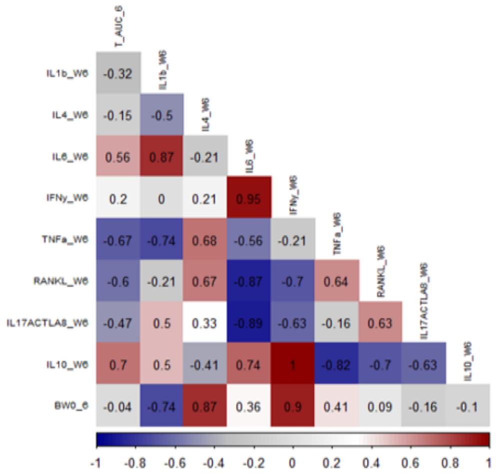

Line IL72

A.

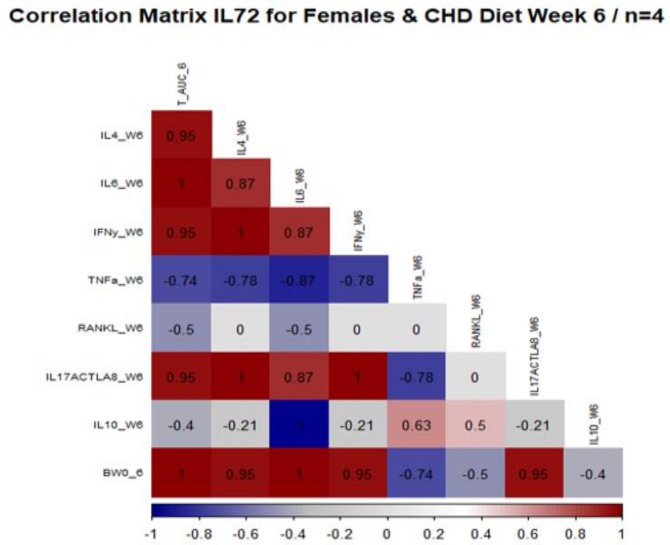

B.

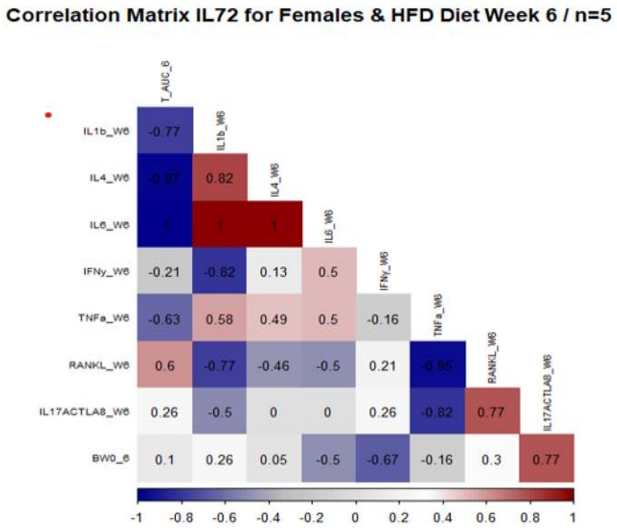

C.

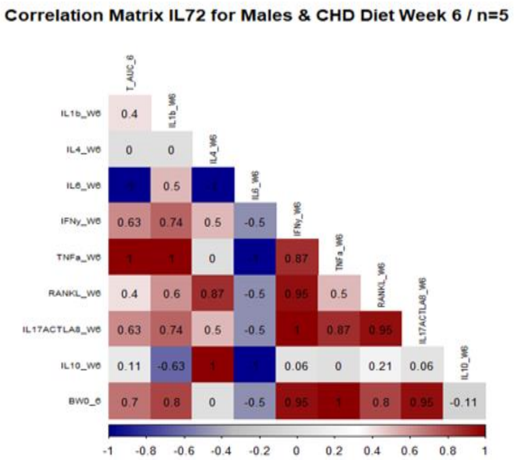

D.

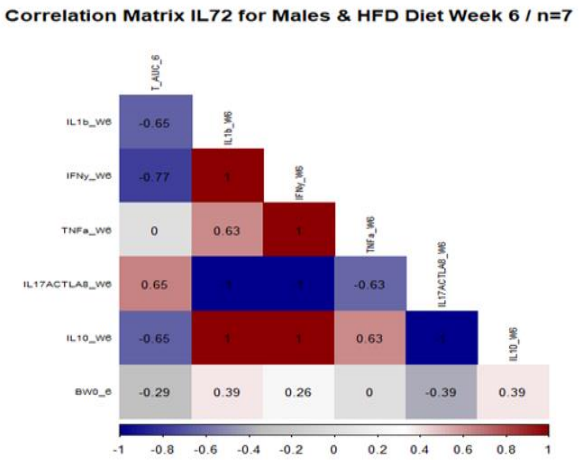

Supplementary Figure 9. Correlation matrix between different inflammatory cytokines, % ΔBWT12, total AUC 12, whole intestine polyps, small intestine polyps, colon polyps' number, and BV for both populations Female and Male. A correlation matrix of three different CC lines (Total mouse population, IL 557, IL711, IL72) of both populations after 12 weeks of diet challenge CHD Vs. HFD intake.

Total mouse population

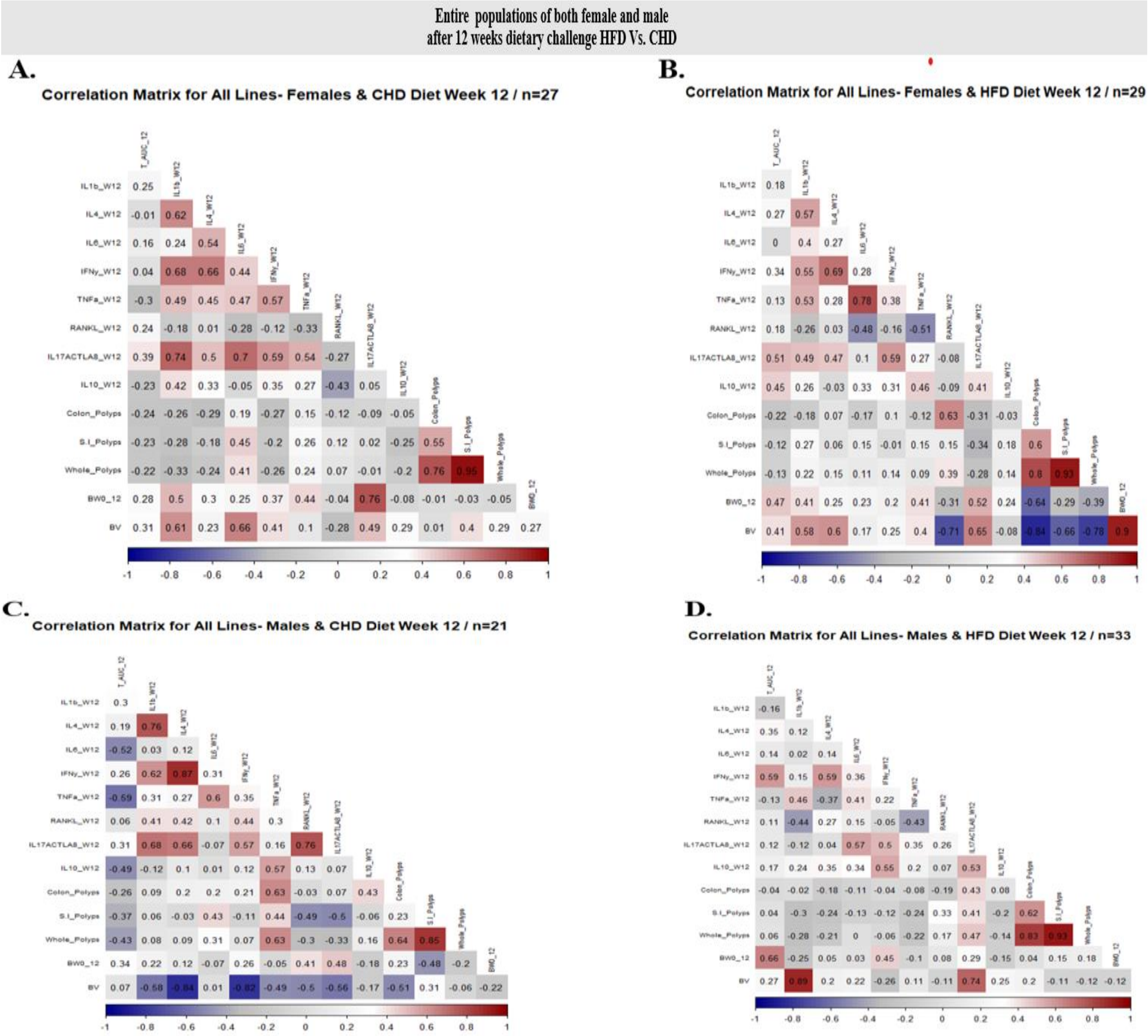

IL557

A.

Correlation Matrix IL557 for Females & CHD Diet Week 12 / n=7

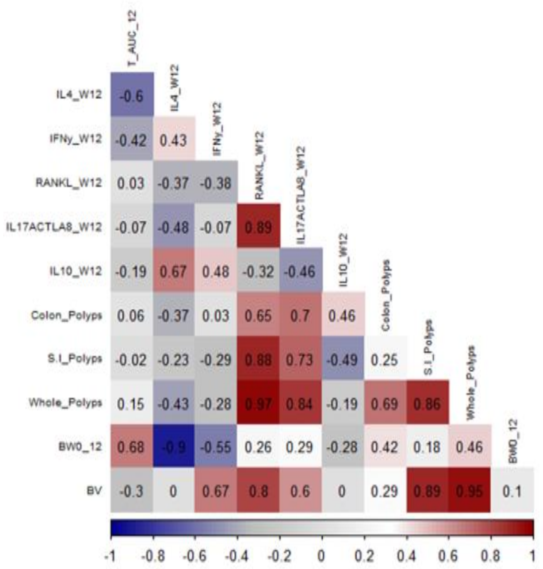

B.

Correlation Matrix IL557 for Females & HFD Diet Week 12 / n=6

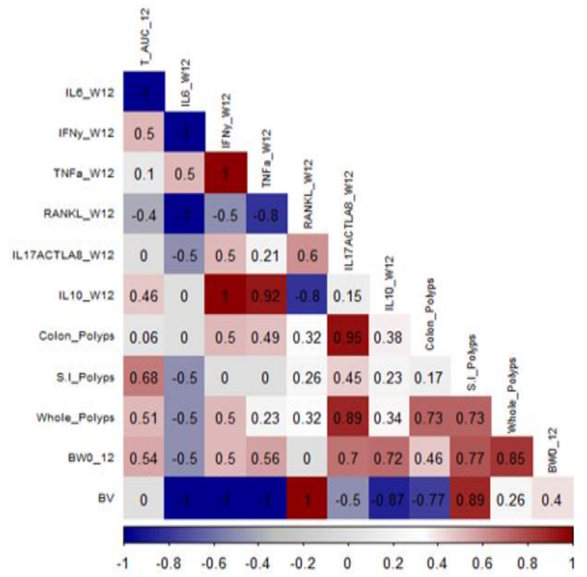

C.

Correlation Matrix IL557 for Males & CHD Diet Week 12 / n=3

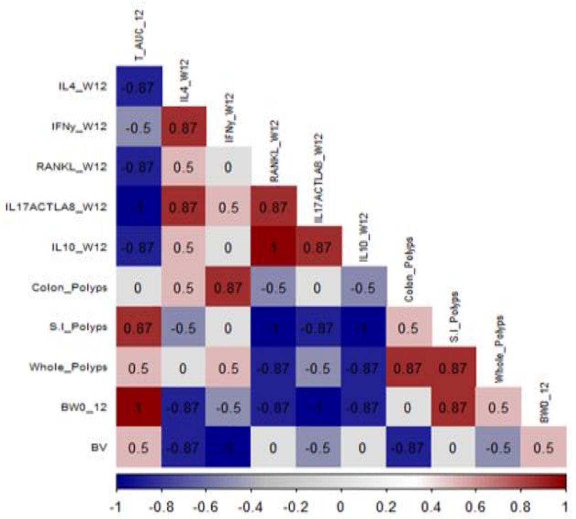

D.

Correlation Matrix IL557 for Males & HFD Diet Week 12 / n=7

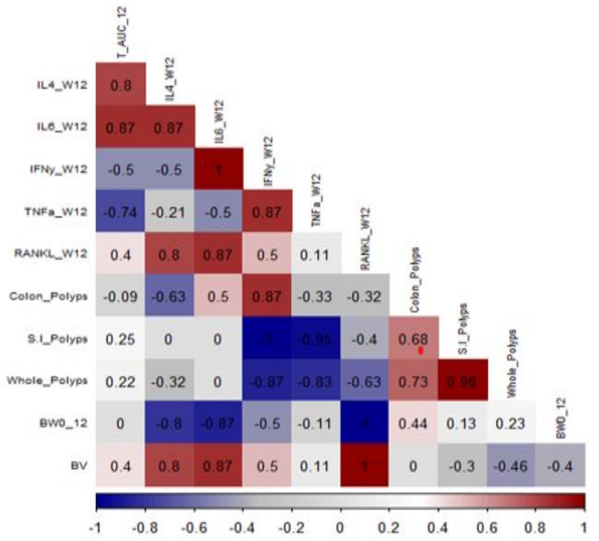

IL72

A.

Correlation Matrix IL72 for Females & CHD Diet Week 12 / n=4

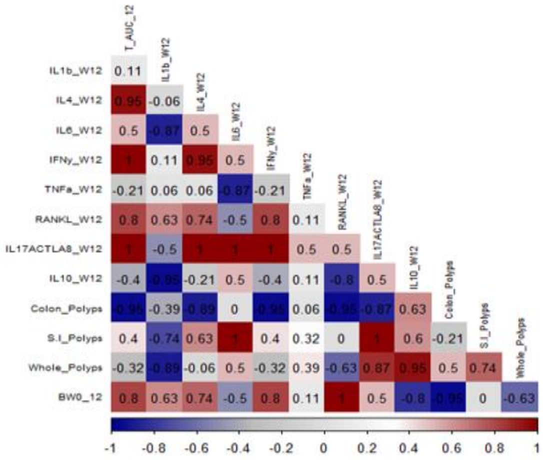

B.

Correlation Matrix IL72 for Females & HFD Diet Week 12 / n=5

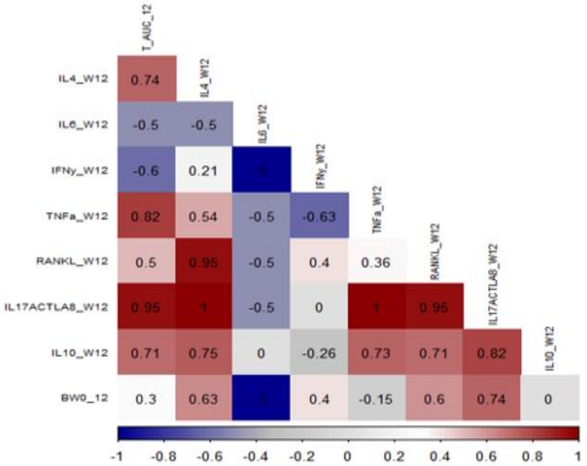

C.

Correlation Matrix IL72 for Males & CHD Diet Week 12 / n=5

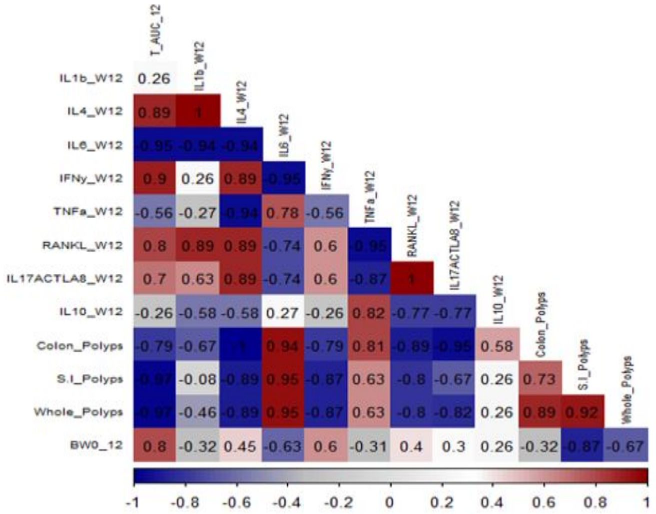

D.

Correlation Matrix IL72 for Males & HFD Diet Week 12 / n=7

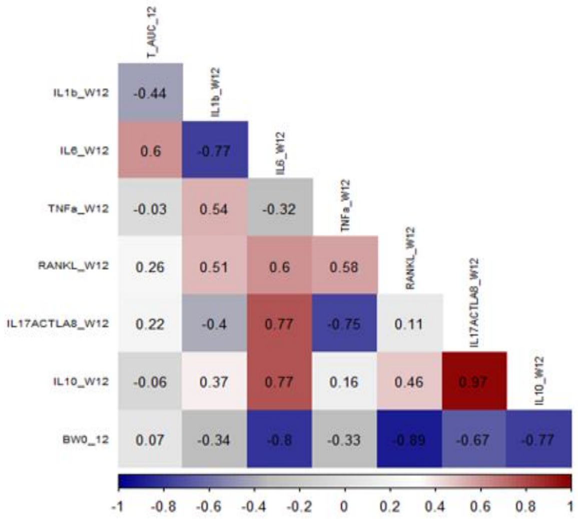

IL711

A.
Correlation Matrix IL711 for Females & CHD Diet Week 12 / n=5

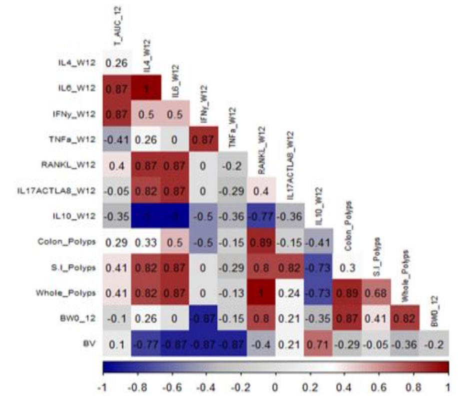

B.
Correlation Matrix IL711 for Females & HFD Diet Week 12 / n=4

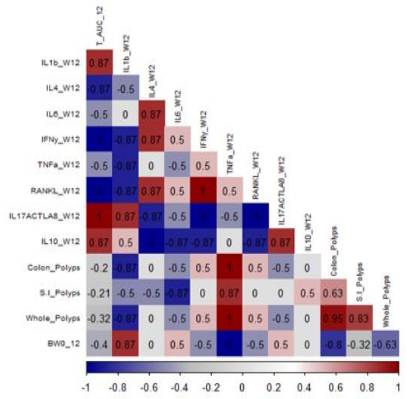

C.
Correlation Matrix IL711 for Males & CHD Diet Week 12 / n=4

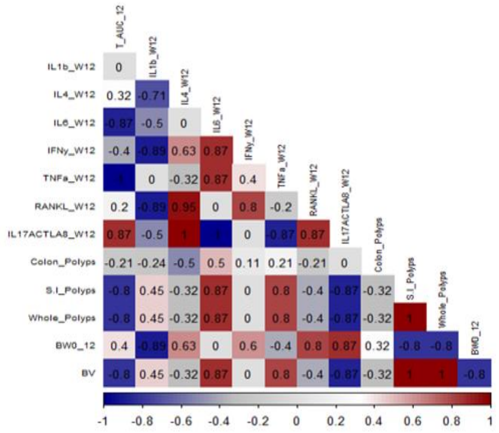

D.
Correlation Matrix IL711 for Males & HFD Diet Week 12 / n=5

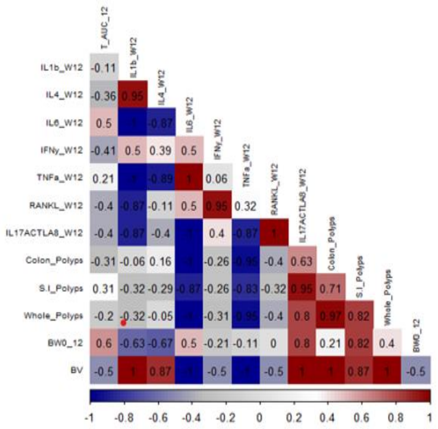

Supplementary Table 1.

A. Summary table shows the list of the used CC lines divided into groups (CHD, HFD) as well as the number of mice of each sex (Female and Male) in small intestine and colon polyp number studies.

		Female		Male		
	CC line	CHD	HFD	CHD	HFD	Total
1	IL72	4	3	3	3	13
2	IL111	2	3	5	2	12
3	IL557	3	8	3	7	21
4	IL711	7	7	4	5	23
5	IL1912	10	7	10	8	35
6	IL2513	5	3	5	9	22
7	IL2750	5	5	8	5	23
8	IL3348	4	2	5	8	19
9	IL3912	3	6	8	5	22
10	IL4141	4	7	8	2	21
11	IL5000	10	17	6	10	43
12	IL5012	3	3	4	3	13
13	IL6009	6	4	3	8	21
14	IL6012	7	7	12	12	38
15	IL6018	6	7	10	8	31
	Total	79	89	94	95	357

B. The summary table shows the list of the used CC lines divided into groups (CHD, HFD) as well as a number of mice of each sex (Female and Male) for Micro CT analysis for PD assessment.

		Female		Male		
	CC line	CHD	HFD	CHD	HFD	Total
1	IL72	3	4	2	3	12
2	IL557	5	5	2	5	17
3	IL1912	9	7	7	3	26
4	IL2513	5	2	3	4	14
5	IL2750	3	2	3	3	11
6	IL3348	4	2	4	3	13
7	IL3912	4	5	6	4	19
8	IL4141	4	3	6	3	16
9	IL5000	3	3	5	3	14
10	IL6018	3	3	4	4	14
11	IL711	5	3	4	3	15
	Total	48	39	46	38	171

C. The summary table shows the list of the used CC lines that were divided into diet groups and the number of mice of each sex (Female and Male) for the correlation matrix of all the traits analysis between the six different traits.

		Female		Male		
	CC line	CHD	HFD	CHD	HFD	Total
1	IL557	5	5	2	5	17
2	IL711	5	3	4	3	15
3	IL2513	5	2	3	4	14
4	IL2750	3	2	3	3	11
5	IL5000	3	3	5	3	14
	Total	21	15	17	18	71

Supplementary Table 2. The body weight changes (%ΔBW) resulted in four different CC lines in the male population due to HFD maintenance for two other time points at weeks 6 and 12 of the experiment.

	CHD	HFD	CHD	HFD
CC line	week 6		week 12	
IL72	26.98	23.20	49.71	22.61
IL111	30.67	11.77	38.21	19.41
IL2513	19.78	10.01	27.72	20.79
IL6018	22.12	11.69	26.51	12.89

Supplementary Table 3. Polyps count ± SE in whole intestine parts of the female population in three different CC lines with a P value < 0.05.

	Female	
CC lines	CHD	HFD
IL2513	3.80±0.489	8.33±1.20
IL5012	5.33±0.881	7.66±0.66
IL6012	2.28±0.359	5.71±0.808

Supplementary Table 4. Represent colon polyps' number in 6 CC lines under dietary challenge CHD Vs. HFD.

	Colon polyps count		
**CC line**	**CHD**	**HFD**	**P value**
**IL72**	**1.1429**	**2.5000**	**0.016**
**IL2513**	**1.0000**	**2.5833**	**0.027**
**IL5000**	**0.6000**	**1.8846**	**0.000**
**IL6012**	**0.7368**	**2.1053**	**0.010**
**IL4141**	**3.4167**	**1.8889**	**0.000**
**IL1912**	**3.2000**	**1.2143**	**0.015**
